# Trustors’ disregard for trustees deciding quickly or slowly in three experiments with time constraints

**DOI:** 10.1038/s41598-022-15420-2

**Published:** 2022-07-15

**Authors:** Antonio Cabrales, Antonio M. Espín, Praveen Kujal, Stephen Rassenti

**Affiliations:** 1 Department of Economics, University Carlos III Madrid, Getafe, Spain; 2 Department of Social Anthropology, University of Granada, Campus de Cartuja S/N, 18071 Granada, Spain; 3 Loyola Behavioral Lab, Loyola Andalucía University, Seville, Spain; 4 Department of Economics, Middlesex University, London, UK; 5 Economic Science Institute, Chapman University, Orange, USA

**Keywords:** Human behaviour, Social evolution

## Abstract

Many decisions in the economic and social domain are made under time constraints, be it under time pressure or forced delay. Requiring individuals to decide quickly or slowly often elicit different responses. Time pressure has been associated with inefficiency in market settings and market regulation often requires individuals to delay their decisions via cooling-off periods. Yet, recent research suggests that people who make reflective decisions are met with distrust. If this extends to *external* time constraints, then forcing individuals to delay their decisions may be counterproductive in scenarios where trust considerations are important, such as in market and organizational design. In three Trust Game experiments (total number of participants = 1872), including within- and between-subjects designs, we test whether individuals trust (more) someone who is forced to respond quickly (intuitively) or slowly (reflectively). We find that trustors do not adjust their behavior (or their beliefs) to the trustee’s time conditions. This seems to be an appropriate response because time constraints do not affect trustees’ behavior, at least when the game decisions are binary (trust vs. don’t trust; reciprocate vs. don’t reciprocate) and therefore mistakes cannot explain choices. Thus, delayed decisions per se do not seem to elicit distrust.

## Introduction

Humans often trust others, but not everyone and not all the time. The extent of trust is instead dependent on the individual and the situation encountered. However, are we able to predict *when* someone can be trusted? There is little doubt that this is a crucial question for understanding human social behavior, organizational performance, and the functioning of societies^1–8^. The outcomes of many decisions in the social domain depend on the behavior of others and we need to form expectations to adjust our behavior accordingly. For this reason, we often gather information not only about our interaction partners’ identity or emotional state^3,5,8,9^, but also about the process through which they make decisions^7,10–13^.

One bit of information that can be an important determinant of trust is the time others have for decision making. That is, are we more likely to trust those that have less time to respond or those that have sufficient time to reflect on their actions? Although the underlying forces are still debated, there is evidence indicating that social behavior is partly driven by the extent to which intuition or reflection dominates the decision process^14–20^. Thus, in a strategic context, it is but natural that we take into consideration whether our interaction partners have sufficient time to reflect upon their choices or not.

Time pressure is indeed ubiquitous in real-life decisions. For example, time-constrained “exploding” offers are frequently used in online consumer markets (see for example eBay, online booking etc.) and are also common in the organization of many other markets (e.g., labor and matching markets^21^). The resulting outcome, however, may be inefficient due to the parties’ reduced opportunity to consider different alternatives^21,22^, which has important implications in both the public and private spheres^23^.

In response to this, regulations that impose “cooling-off” periods are often set in place with the expectation that delaying decisions will presumably lead to more efficient outcomes^24–27^. But forcing individuals to delay their decisions may lead to unintended consequences if the reflective (vs. intuitive) character of the decisions per se influences other parties’ perceptions, hence triggering differential behaviors. One key consideration in markets and other types of social interactions is, in fact, trust. Recent behavioral evidence suggests that people who reflect upon their decisions are met with distrust^6,7,10^. If this extends to reflective decisions per se, then forcing agents to delay their decisions may have a detrimental effect on trust and thus be ineffective or even counterproductive in terms of efficiency. However, whether individuals adjust their trust to their counterparts’ time constraints remains understudied.

We shed light on this issue by studying a canonical example of strategic interaction where adjusting to others’ behavior is key to reach optimal outcomes. We conduct a series of one-shot Trust Game^28^ (TG) experiments in which a “trustor” can send a certain amount of her endowment to a “trustee” and is informed about the time constraint under which the trustee is making her decision. The trustee, on the other hand, can reciprocate (or not) the trust placed in her either quickly, in one condition, or after a delay, in the other. The trustor’s final payoff depends crucially on trustee’s trustworthiness. If the trustee is trustworthy enough, then the trustor maximizes her payoff by sending the entire endowment. This is the socially efficient outcome. Yet, full trust is risky and leaves the trustor vulnerable to receiving nothing.

In our experiments, trustors were informed that trustees would have to make their decisions either within a time limit of 10 s (i.e., the *time pressure* condition) or after 10 s have elapsed (i.e., the *time delay* condition). According to the evidence we review in what follows, we predict that in our experiments:

H1. Trustors will display *greater trust in the time pressure condition* than in the time delay condition.

H2. The mechanism causing H1 is that trustors *expect trustees to be more trustworthy under time pressure* than under time delay.

While the effect of forcing fast versus slow decision making in social interactions has been extensively studied^14,15,18,29–31^, less research has been conducted to understand whether people correctly anticipate such an effect and adjust their behavior accordingly. This seems to be an important issue for all social decisions involving strategic uncertainty (besides our canonical TG implementation).

The available evidence suggests that people who make “calculated” decisions may be met with distrust by others. Specifically, those individuals who (on their own) deliberate upon their choices, either by looking carefully at the payoffs or by delaying their decisions, appear to be perceived as less prosocial^13^ (but see ref.^32^), and are trusted less^7^. This occurs even when calculated and uncalculated decisions are equally prosocial^7^. The moral character of people who make (moral) decisions quickly is also rated more positively than that of people who make them slowly, even if their final decisions are identical^10^. Moreover, people who express deontological moral judgments, which are thought to be less calculated than consequentialist/utilitarian judgments^33^, are trusted more^6,8,34^. Interestingly, people seem to anticipate this effect and tend to reflect less upon their cooperative decisions when potential interaction partners are observing compared to when they are not^7^. This is consistent with uncalculated cooperation being used as a signal of trustworthiness, which may indeed serve a long-run self-interested (fitness-maximizing) goal^12^.

The studies reviewed above, however, are based on endogenous decisions and therefore responses to the decision-making process itself cannot be separated from responses to the inferred moral character of the decision maker. That is, it is unclear whether people attach a greater positive value to (i.e., they trust more) less reflective decisions when the reflective versus intuitive character of decisions is *externally imposed* rather than being the outcome of an endogenous process. If this is the case, then the effects of the decision maker’s cognitive mode on others’ behavior may not (only) be related to inferences about her underlying disposition but also to beliefs about the consequences of reflection itself. In other words, do individuals distrust those who reflect, or reflective decisions per se? Here we focus on the latter question. An affirmative answer would challenge previous claims that people who make reflective decisions are met with distrust because they are seen as less moral: at least part of the effect might be due to others’ perception about the pure effect of reflection versus intuition, regardless of the decision maker’s inferred character.

Recent experiments suggest that “emotion” is perceived by partners to trigger more cooperative behavior than “reason” when emotion-based versus reason-based decision making is externally induced, and indeed partners cooperate more in the former case^35^. While emotion is often associated with intuition, reason with reflection^35,36^ and cooperation with trust^37,38^, whether this result can be extended to the speed of decision making and to pure trust situations remains unknown.

Several previous results indeed suggest that intuitive (vs. reflective) decision making may trigger more trustworthy, prosocial behavior in one-shot interactions^14,15,30,39,40^ (see ref.^20^ for a review), and there exist evolutionary reasons why this should be the case^41,42^. These findings imply that the trustors’ hypothesized adjustment in beliefs and behavior is optimal, in the sense that trustees will in fact be more trustworthy under time pressure compared to time delay. However, other studies indicate that the effects of time constraints on social behavior are complex and depend on a set of factors including individual heterogeneity, the presence of mistakes, previous experience in similar experiments, the weights of different distributional motives, and the specific social environment individuals regularly face^16,18,29,43–46^. In our experiments, we also study trustees’ behavior and report the results for completeness. Given the mentioned complexity and mixed results, however, we do not set any hypothesis about trustee’s behavior.

The only other study, to our knowledge, that elicited individuals’ beliefs to external time constraints has been conducted in ref.^32^. They used similar time constraints to ours in a Public Goods Game and found that fast decisions are not expected to be more, nor less, cooperative than slow decisions when time constraints are externally imposed. As they elicited beliefs towards a “hypothetical” person, it was not possible to check whether they were correct or biased. In any case, the TG and the Public Goods Game elicit different social behaviors, being (beliefs about) others’ decisions especially important in the former: trusting is payoff-maximizing for the trustor if the trustee is trustworthy enough in the TG, while cooperation is never payoff-maximizing in the Public Goods Game regardless of other players’ decisions.

In our TG experiments, we used three different designs. In Study 1 (*n* = 300, US university students), both players started with an endowment of $10. Roles were randomly assigned. The trustor, moving first, could send any amount between $0 and $10 (in $0.01 increments) which would then be tripled before reaching the trustee. Finally, the trustee had to decide which part of the received amount (i.e., 3 × *trusted amount*) she wanted to return to the trustor. Thus, in an “ideal” scenario in terms of social efficiency and equity of outcomes, the trustor would send the entire endowment and the trustee would return exactly half of the total amount resulting in a payoff of $20 for both players. However, in the case of being fully trusted, an untrustworthy trustee can take home $40 leaving the trustor with nothing. In our experiment, one half of the playing pairs were randomly assigned to the *time pressure* or *time delay* conditions (referring to the trustee’s time constraints; that is, the trustor decided under no time constraints in all our studies).

In Study 2 (*n* = 795, US MTurk workers), we simplified the design to facilitate both the trustee’s decision making and the elicitation of the trustor’s expectations about the trustee’s trustworthiness. This design also minimized the possibility that the trustees could think ex-ante upon their decision (see below). We used a binary TG in which the trustor could decide to send either her whole endowment ($0.40) or nothing to the trustee, who also starts with $0.40. The money sent was tripled. The trustee had to decide whether to send $0.80 back to the trustor and keep $0.80 or to keep all the $1.60 for herself in case of being trusted. Before learning the outcome, participants were asked to guess the average response of both players, that is, the percentage of trustors who chose to trust and the percentage of trustees who chose to be trustworthy. Correct responses (i.e., accurate beliefs) received an additional bonus payment. One half of the playing pairs were randomly assigned to either the *time pressure* or *time delay* conditions.

In Study 3 (*n* = 777, US MTurk workers, different from those in Study 2), we implemented a within-subjects design (only for trustors). Using the same action and payoff structure as in Study 2, we asked the trustors to make two trust decisions, one for each time condition, in random order. This allows trustors to compare both conditions very easily and adjust their behavior accordingly. Beliefs were also elicited for the two conditions in random order.

The results are clear cut: across all the three studies, we fail to find any robust effect of trustees’ time constraints on trustors’ decisions or beliefs. Given that trustees’ behavior, at least in the cleanest designs of Study 2 and 3 (which minimize the effect of mistakes and the possibility of deciding ex-ante), is not affected by external time constraints in our experiments, trustors’ response seems accurate.

Below we report on the methods and the results for  each study for the effect of the (trustees’) time conditions on both trustors’ and trustees' decisions. Our main research question  constitues of H1 and H2 which are regarding trustors. The last section concludes with a general discussion.

## Study 1

A total of 300 students (63% females) from Chapman University in the US participated in our experiments. Some of the results from this study were circulated in a previous working paper^47^. These participants were recruited from a database of more than 2000 students. A subset of the whole database received invitations at random for participating in the current study. The local IRB approved this research, which was conducted in accordance with relevant regulations and the Declaration of Helsinki. All participants provided informed consent prior to participating. No deception was used. Participants were paid the amount earned during the experiment (mean ± SD = $14.73 ± 6.75) plus a $7 show-up fee.

We conducted 20 sessions with a minimum of 6 and a maximum of 22 participants. Sessions lasted for approx. 30 min. Participants were randomly assigned to either a time pressure or a time delay session (*n* = 150 in each condition) and subsequently to either the trustor (labeled as “individual A”) or the trustee (“individual B”) role of the Trust Game^28^. Thus, we collected data from 75 participants in each condition/role. Following ref.^31^, this sample size was determined a priori to detect a medium size effect (Cohen’s *d* = 0.50) with 85% power and alpha = 0.05, two-tailed: minimum *n* = 73 in each condition/role. Participants were unaware of the existence of another experimental condition. All procedures were computerized.

Upon arrival to the laboratory, subjects were randomly assigned to cubicles (which impeded visual contact between them) and were randomly matched in pairs with another anonymous participant  who was assigned the other role. Subsequently, the instructions for their specific role were displayed on the computer screen. Participants in both roles started the game with $10. Before learning the rules of the game, subjects familiarized themselves with the image and the pointer of the decision slider (without any values on it). This was done to reduce potential mistakes, especially by trustees in the time pressure condition. However, this familiarization might allow trustees to make ex-ante (loose) inferences about the decision they would have to make, thus potentially biasing the effect of time constraints. Trustors were then asked which part of their $10 they wanted to send to the trustee and were informed that the trustee would receive three times the amount transferred. Trustees were subsequently asked to decide what proportion of the amount received to return to the trustor. In the time pressure [delay] condition, participants in both roles were informed that *trustees* had to make their decision before [after] a 10-s timer expired.

All these instructions were common knowledge. In both conditions, trustees saw the timer on the screen counting down from 10 to 0. An identical slider bar was used by all participants to decide how much money to transfer to the other party and how much to keep by clicking on the desired point of the slider (in $0.01 increments). For trustors, the maximum amount to transfer was $10, whereas for trustees the amount to share was three times the amount received. All trustees respected the time constraints; otherwise, they would not be allowed to make their decision (which implies that both players would earn only the $10 endowment). Average (± SD) response time among trustees was 7.79 s (± 2.37) in the time pressure condition and 32.70 s (± 14.10) in the time delay condition.

After playing the TG, all participants completed a questionnaire in which we assessed their (1) risk preferences using a multiple-price-list lottery task^48^, (2) distributional social preferences using a series of mini-dictator games^16,49^, (3) time preferences using a multiple-price-list intertemporal choice task (adapted from ref.^50^), and (4) cognitive styles using an extended version of the Cognitive Reflection Test^51,52^. Participants were paid an extra fixed amount of $3 for responding to the questionnaire and were unaware of its existence prior to playing the TG.

Full experimental instructions, including those of all the tasks included in the questionnaire which are used in this study, can be found in Appendix A1 in the supplementary materials.

### Results study 1: trustors

**Figure 1:**
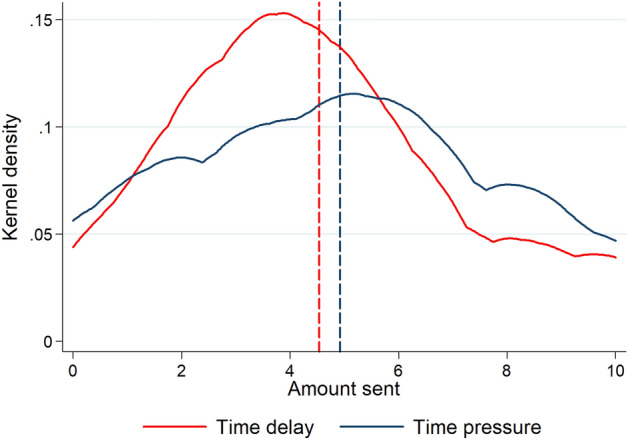
Kernel density estimation for amount trusted in the time delay (solid red line) and time pressure (solid blue line) conditions. Dashed vertical lines depict means (time delay: red line, time pressure: blue line). Study 1.

Figure 1 displays the Kernel density estimation for the distribution of amount trusted for the two conditions (*n* = 75 in each condition). Although the effect is in the predicted direction (H1), there are no significant differences in average trust (OLS regression with robust standard errors [from now on standard errors are always robust]: coeff of *time delay* = − 0.378, *p* = 0.42, *n* = 150). Mean (± SEM) amount sent: time pressure = 4.92 ± 0.35, time delay = 4.54 ± 0.31, which stands for a Cohen’s *d* of 0.13. The median amount sent is $5 and $4, respectively. Neither the Mann–Whitney nor Kolmogorov–Smirnov distribution tests yield significant differences between conditions (*p* = 0.39 and *p* = 0.45, respectively). However, as can be seen from Fig. 1, the amount sent by trustors in the time delay condition is concentrated between $2 and $5, whereas in the time pressure condition the distribution is flatter. Indeed, the likelihood of sending an amount between $2 and $5 is significantly higher under time delay compared to the time pressure condition (probit regression with robust standard errors: mfx of *time delay* = 0.200, *p* = 0.009, *n* = 150). The regression analysis can be found in supplementary Table S1, model 2a. Thus, we find no support for H1, but our results suggest that trust levels are more concentrated in low-to-medium values in the time delay condition as compared to the time pressure condition.

As shown in Table S1 (models 1b and 2b), all these results are robust to controlling for the decision maker’s gender^51,53–55^, CRT score^16,18,45^, distributional social preferences^16,18,56,57^, time preferences^50,58,59^, and risk preferences^56,60^, that could constitute confounding factors (as randomization could lead to imperfect balance in relatively small samples).

### Results study 1: trustees

**Figure 2:**
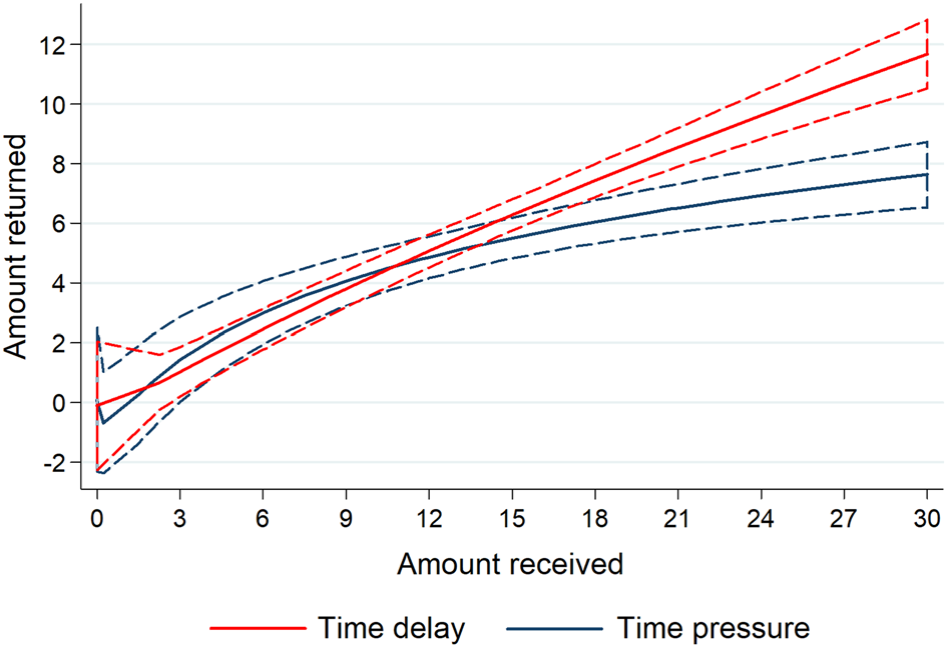
Fractional polynomial plot of amount returned as a function of amount received in the time delay (solid red line) and time pressure (solid blue line) conditions. Dashed lines depict 90% CI. Study 1.

In Fig. 2, we present the amount returned (± 90% CI) in the two conditions as a function of the amount received in Study 1. We plot this effect using fractional polynomial analysis which allows us to capture complex non-linear relationships. It can be observed that for amounts received above $15 (i.e., amount trusted = $5), trustees in the time delay condition seem to return higher amounts than in the time pressure condition. While the positive relationship between amount returned and amount received apparently displays some concavity in the time pressure condition, using OLS estimation we cannot reject that the relationship is linear (i.e., not concave; a regression with *amount received* and *amount received squared* as explanatory variables yields *p* > 0.19 for *amount received squared* in both conditions). Thus, we run OLS regressions in which amount received is assumed to have a linear effect on amount returned.

First, we conduct a main effects analysis with the amount received and condition as explanatory variables: both yield significant estimates (coeff of *amount received* = 0.309, *p* < 0.001, *n* = 150; coeff of *time delay* = 1.081, *p* = 0.026). Second, we analyze the interaction between the two variables, which is also significant (coeff of *time delay* × *amount received* = 0.149, *p* = 0.003, *n* = 150). This tells us that in the time delay condition the amount returned increases significantly more with the amount received relative to the time pressure condition (coeff *of amount received*: in time pressure condition = 0.245, *p* < 0.001; in time delay condition = 0.394, *p* < 0.001; Wald tests on the interaction model coefficients). The regression analyses can be found in Table S4, model 1a and 2a, respectively. Adding controls does not change the results (models 1b and 2b).

According to the model estimates, the amount trustees return is not significant in the time delay condition (*p* > 0.07 for all trusted amounts of $4 or less; Wald tests on the interaction coefficients). For trustees who were sent $5 (i.e., who received $15), the model reports that the amount returned is significantly higher in the time delay compared to the time pressure condition ($6.09 and $4.88, respectively; *p* = 0.015, Wald test). The largest difference is estimated for trustees who were trusted with the whole endowment (i.e., those receiving $30; $12.00 and $8.55, respectively; *p* = 0.002, Wald test). This is consistent with the observation from Fig. 2.

### Discussion of study 1

The results of Study 1 do not provide clear support for our initial hypothesis (H1) which states that the time delay condition should trigger more distrust than the time pressure condition. Interestingly, ref.^32^ found that individuals expected fast decisions to be more extreme (i.e., either full defection or full cooperation) than slow decisions in a Public Goods Game, although not significantly so when response times were externally imposed. Similar expectations of extremity might have attenuated the (hypothesized) detrimental effect of time delay vs. pressure on expected trustworthiness—and on trust as a result—and might help explain why extremely low trust levels (below $2) are not more likely to arise in the time delay condition. Yet, we did not elicit trustors’ expectations in this study.

## Study 2

In Study 2, apart from studying a more heterogeneous sample, we address some of the limitations of Study 1. On the one hand, Study 2 has a larger number of observations which alleviates concerns about lack of statistical power. On the other hand, we implement a binary Trust Game (i.e., trust vs. don’t trust; reciprocate vs. don’t reciprocate) rather than a continuous one. This design feature allows us to alleviate several concerns that could arise in Study 1. First, since there are only two possible actions, it eliminates the potential effect of expectations of extremity^32^ which might have reduced the hypothesized effect.

Another mechanism that can reduce the hypothesized effect is trustors’ expectation that under time pressure trustees can make more errors^36,46^, thus returning a small amount by mistake after being trusted a large amount. If trustors fear that trust might not be reciprocated due to error, even if trustees are expected to be more trustworthy under time pressure, then reducing trust would be a good strategy to alleviate the risk. Of course, lower trusted amounts leave less room for trustees’ mistakes, so this might artificially reduce trust levels in the time pressure condition when the decision space is continuous and counteract the effect of a higher expectation of trustworthiness under time pressure.

Third, due to the simplicity of the binary game the instructions for the experiment are greatly simplified and trustees do not need to familiarize with the decision screen. This is important as they cannot deliberate their decision in advance. Finally, in the continuous TG it is difficult to assess trustors’ beliefs about trustees’ trustworthiness. Indeed, we did not elicit trustor’s expectations in Study 1. Note that trustors in Study 1 had 1001 possible actions (i.e., trusted amounts could range between $0 and $10 in $0.01 increments). Elicitation of beliefs in this case needs to reduce the decision space for practical reasons (e.g., to intervals of trusted amounts, or to the trustor’s actual trust level), which may induce decision-making biases and information losses. In addition, a low (proportional) back-transfer does not have the same connotations when it comes from a low trusted amount, which might be a signal of negative reciprocity, as when it comes from a high trusted amount, which is a clear signal of untrustworthiness. This fact complicates designs in which other players need to consider trustees’ decisions to make their choices^61^. The binary structure of the TG in Study 2 will allow us to elicit trustors’ beliefs in a straightforward and reliable manner.

Compared to Study 1, Study 2 allows us to get a clearer picture of the behavior of both trustors and trustees in each condition, to study trustors’ beliefs, and to alleviate concerns about lack of statistical power and sample heterogeneity.

A total of 795 US-settled individuals (after excluding duplicate IPs, as standard; 53% females) recruited from Amazon Mechanical Turk^62,63^ (MTurk) participated in the experiment. The IRB of Middlesex University approved this research, which was conducted in accordance with relevant regulations and the Declaration of Helsinki. The participation fee was $1 for a 10–15 min experiment. In addition, participants could earn extra money depending on their decisions and/or those of others during the experiment. The average (± SD) bonus was $0.69 ± 0.50.

Participants first entered a valid MTurk ID in the Qualtrics surveyand were then randomly assigned to the role of trustor (labeled as “player A”) or trustee (“player B”), on the one hand, and to the time pressure (*n* = 197 trustors, *n* = 200 trustees) or time delay condition (*n* = 200 trustors, *n* = 198 trustees), on the other. For comparability with the continuous TG of Study 1, this sample size allows us to detect a small-to-medium effect size (Cohen’s *d* = 0.30) with 85% power and alpha = 0.05, two-tailed. Translated into the binary case, this sample size allows us to detect relatively small proportion differences (between 0.05 and 0.15 approx., depending on the specific proportions) with 85% power and alpha = 0.05, two-tailed. Trustors and trustees within each condition were randomly matched to calculate their payments. The decisions of three (randomly selected) trustees in the time pressure condition and two trustors in the time delay condition were used to calculate payments for unmatched participants.

The Trust Game was implemented as follows. The trustor had to choose between “option R” (not to trust) and “option L” (trust). Option R allocated $0.40 to the trustor and $0.40 to the trustee. If the trustor chose option L, the trustee had to choose between options Y (not to reciprocate) and X (reciprocate). Option Y allocated $1.60 to the trustee and $0 to the trustor, whereas option X allocated $0.80 to both players. Note that this design resembles a TG in which the trustor can send a $0.40 endowment to the trustee, and the money trusted is then tripled before reaching the trustee (i.e., the trustee receives $1.20), who has to choose whether to keep all the $1.60 (i.e., $1.20 + the $0.40 endowment) or send $0.80 in back to the trustor. In contrast to Study 1, trustees decided under the so-called strategy method^64^ (applied to the binary TG, for example, in ref.^57^). That is, they had to decide whether to reciprocate or not (i.e., to choose option X or Y) ex-ante, without knowing if the randomly matched trustor will trust (option L) or not (option R); in the latter case, obviously, the trustee’s decision would have no consequences (see instructions in Appendix A2).

The time conditions were the same as in Study 1. Trustees had to choose between options X and Y either before or after a 10-s timer elapsed, which refer to the time pressure and time delay conditions, respectively. Before reaching the decision screen, the trustees knew the payoffs in case the trustor chose option R (i.e., $0.40 each), and that if the trustor chose option L they had to choose between options X and Y, but they did not know anything about the consequences of options X and Y, so that they could not think of their decision in advance. Importantly, trustees did not need to familiarize with the task to avoid mistakes because their decision was extremely simple, in contrast to Study 1. Neither the trustor nor the trustee knew about the existence of another time condition. Average (± SD) response time among trustees was 5.75 s (± 4.69) in the time pressure condition and 11.51 s (± 12.79) in the time delay condition. Non-compliance with the time constraint assigned, as in Study 1, implied that the interaction would not take place and both players earn $0.40 (as if the trustor chose not to trust). This was known in advance by the participants. Still, 6% of the trustees in the time pressure condition and 39% in the time delay condition failed to comply. The latter figure may be influenced by the fact that, to check compliance, we used the last time the participant clicked on the option chosen (X or Y) rather than the time when they clicked on the button to send their decision; the instructions explicitly mentioned this. Using the “send” click, non-compliance is still high but is reduced to 30%. Since our participants are MTurk workers, one likely explanation for low compliance in the time delay condition is that they want to finish early. This is a job after all and some of them may not be seeing the reason for waiting (i.e., earning some extra money) sufficiently encouraging. To avoid that non-compliance biases our results, we analyze the behavior of both compliant and non-compliant individuals, as standard^31^.

After they made their decisions (without feedback), all participants were asked to guess the percentage of player A's choosing option L and the percentage of player B's choosing option X (both only for their own time condition), in this order. Guesses were implemented in 5% increments from 0 to 100% and were considered correct if the actual percentage belonged to the interval “guess ± 5%”. Participants received $0.10 for each correct guess. Participants were correct only 13% of the time and the earnings were $0.026 (SD 0.046) out of a maximum of $0.20.

Finally, the participants had to complete the same risk and distributional social preferences tasks as in Study 1, as well as the 7-item CRT (to avoid that the participants could find the CRT answers online, we implemented the adapted version from ref.^18^, which slightly modifies the wording and answers of the original tasks in refs.^51,52^ in order to be applied in online settings), but in this case the tasks were incentivized in a probabilistic manner. We chose to incentivize the CRT with $0.30 for each correct answer in order to ensure considerate responding, which is especially important in online experiments. In addition, they were asked to complete a loss aversion task^65,66^ which allows us to control for risk preferences more robustly, in particular, in the domain of losses. One out of every 10 participants were randomly selected to receive the real payment associated with one randomly selected task among the four (probabilistic payments have been proven to provide valid data in economic experiments^67,68^). Given the difficulty of implementing delayed payments in MTurk experiments and that previous literature finds no difference between hypothetical and real choices in time preferences tasks^69–71^, the elicitation of the participants’ time preferences was implemented using hypothetical rewards. Full instructions can be found in Appendix A2.

### Results study 2: trustors

**Figure 3:**
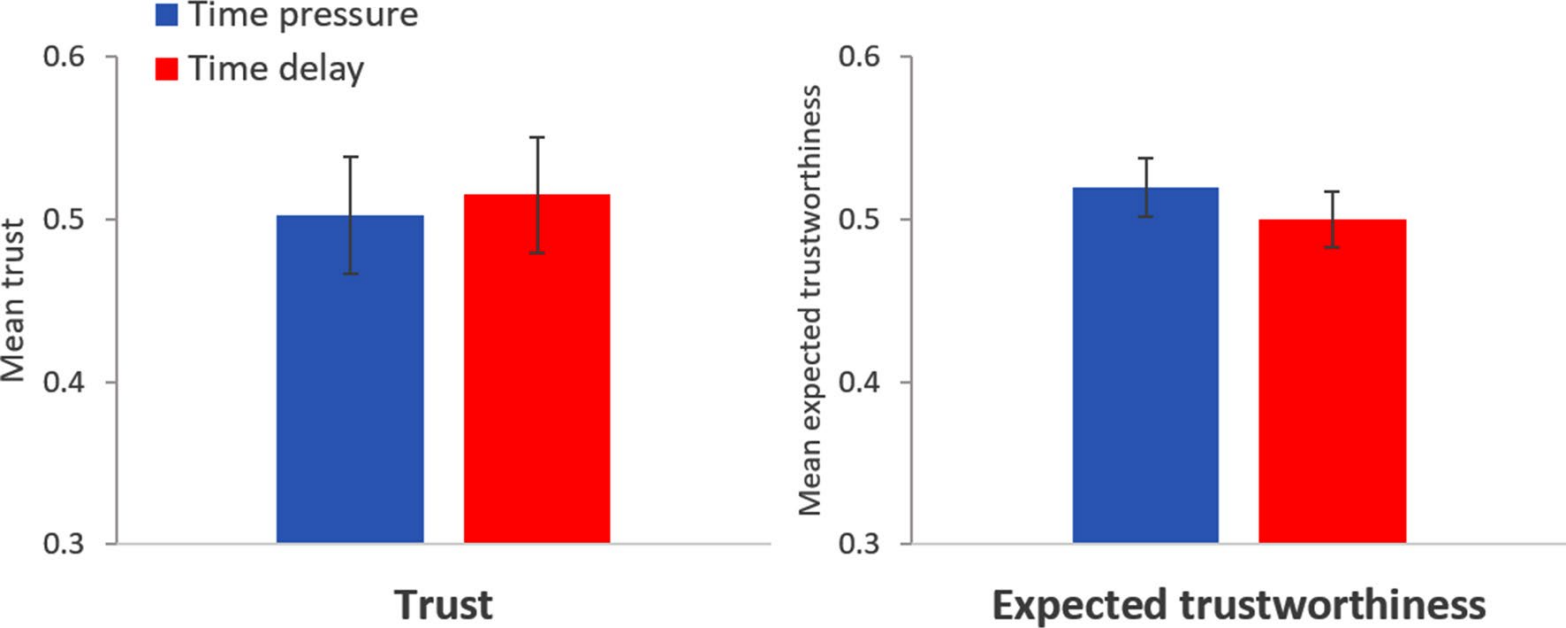
Left panel: Proportion of trustors choosing to trust in the time pressure (blue bar) and time delay condition (red bar). Right panel: Mean trustworthiness expected by trustors in the time pressure (blue bar) and time delay condition (red bar). Error bars represent robust SE. Study 2.

The left panel in Fig. 3 displays the proportion (± SE) of trustors choosing to trust in each condition (*n* = 197 for time pressure; *n* = 200 for time delay). There are no significant differences in trust between the two conditions (probit regression with robust standard errors: mfx of *time delay* = 0.012, *p* = 0.80, *n* = 397), in contrast to H1. Observed proportions (± SE): time pressure = 0.503 ± 0.036, time delay = 0.515 ± 0.035. The regression analysis can be found in Table S2, model 1a. Thus, we find no support for H1.

The right panel in Fig. 3 displays the mean (± SEM) trustworthiness expected by trustors in each time condition, expressed as the (expected) proportion of trustworthy trustees. There are no significant differences in expected trustworthiness between the two conditions (OLS regression with robust standard errors: coeff of *time delay* = − 0.020, *p* = 0.42, *n* = 397), in contrast to H2. Average expected trustworthiness (± SEM): time pressure = 0.520 ± 0.018, time delay = 0.500 ± 0.017. This difference yields a Cohen’s *d* of 0.08. The regression analysis can be found in Table S2, model 4a. As can be seen in Table S2, models 2a and 3a, expected trustworthiness predicts trust both in the time pressure (mfx of *expected trustworthiness* = 0.474, *p* = 0.001) and in the time delay condition (mfx of *expected trustworthiness* = 0.450, *p* = 0.003). In Table S2, models 1b–4b, we show that these results are robust to controlling for all the variables considered in this study. Thus, we find no support for H2, although our results show that expected trustworthiness predicts trust.

### Results study 2: trustees

**Figure 4:**
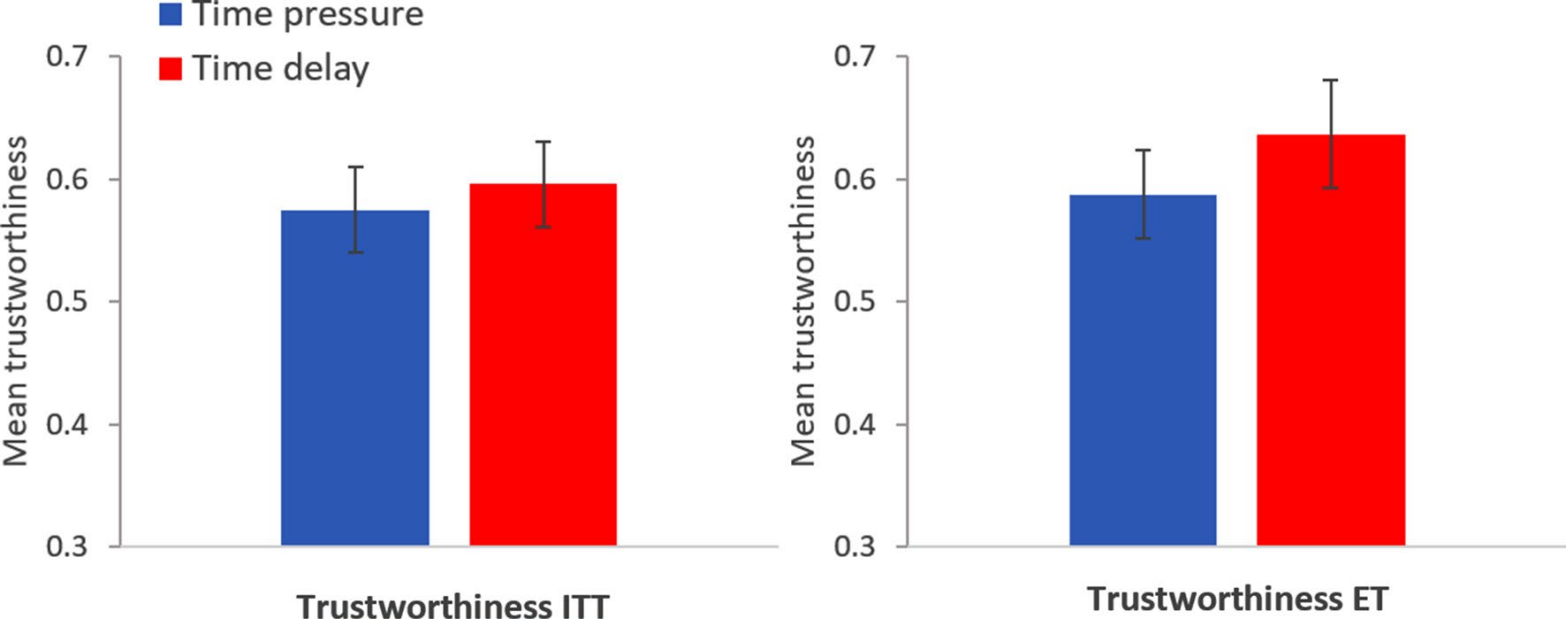
Left panel: Mean proportion of trustworthy trustees in the time pressure (blue bar) and time delay condition (red bar) using an intent-to-treat approach. Right panel: Mean proportion of trustworthy trustees in the time pressure (blue bar) and time delay condition (red bar) using only effectively treated subjects. Error bars represent robust SE. Study 2.

The left panel in Fig. 4 displays the proportion (± SE) of trustworthy trustees in each condition in Study 2 using an intent-to-treat approach^31^ (ITT), in which subjects who did not respect the time condition are included (*n* = 200 for time pressure; *n* = 198 for time delay). There are no significant differences in trustworthiness between the two conditions (mfx of *time delay* = 0.021, *p* = 0.67, *n* = 398). Observed proportions (± SE): time pressure = 0.575 ± 0.035, time delay = 0.596 ± 0.035. The regression analysis can be found in Table S5, model 1a. The right panel in Fig. 4 displays the proportion (± SE) of trustworthy trustees in each condition in Study 2 using only subjects who were effectively treated^14,31^ (ET), that is, those who respected the time condition (*n* = 189 for time pressure; *n* = 121 for time delay). There are no significant differences in trustworthiness between the two conditions (mfx of *time delay* = 0.049, *p* = 0.39, *n* = 310). Observed proportions (± SE): time pressure = 0.587 ± 0.036, time delay = 0.636 ± 0.044. The regression analysis can be found in Table S5, model 2a. In Table S5, models 1b and 2b, we show that these results are robust to controlling for all the variables considered in this study.

### Discussion of study 2

Using a larger sample and a simpler design as compared to Study 1, in Study 2 we again failed to find any effect of the trustees’ time condition on trustors’ trust decisions. In addition, we also find no difference between conditions in expected trustworthiness. These results are thus against our hypotheses H1 and H2. As hypothesized, however, trustors’ trust increases with expected trustworthiness. The latter suggests that the data are reliable.

Still, the between-subjects designs employed in Studies 1 and 2 might have obscured the potential effect of trustees’ time condition on trust. It might be that trustors need to be aware of the existence of both time conditions to be able to adjust their behavior and beliefs. At least, this seems to favor the emergence of differences between conditions. In Study 3, we implement a within-subjects design to address this question. This design will also allow us to obtain results with greater statistical power.

## Study 3

Study 3 aims to alleviate concerns related to the argument that trustors might need to compare both conditions to adjust their behavior and beliefs. Thus, we implement a within-rather than between-subjects design, which also provides for an analysis with greater statistical power. This design brings a scenario which seems to be very favorable for any difference between conditions to emerge and, therefore, to find support for our initial hypotheses.

A total of 777 US-settled MTurk workers (after excluding duplicate IPs; 46.5% females) participated in the experiment. The IRB of Middlesex University approved this research, which was conducted in accordance with relevant regulations and the Declaration of Helsinki. Participants from Study 2 were excluded. The participation fee was $1 for a 10–15 min experiment. In addition, participants could earn extra money depending on their decisions and/or those of others during the experiment. The average (± SD) bonus was $0.72 ± 0.49.

After entering a valid MTurk ID in the Qualtrics-based survey, the participants were randomly assigned to the role of trustor (*n* = 376) or trustee (*n* = 401). Those in the role of trustor (“player A”) would be asked later on to make one decision for each time condition (see below): they were randomly assigned to choosing first either for the time pressure case (*n* = 183) or for the time delay case (*n* = 193). This sample size allows us to detect a small effect (Cohen’s *d* = 0.17) with 85% power and alpha = 0.05, two-tailed, for a within-subjects design (assuming a correlation of 0.4 between the two measures). Translated into proportions, this sample size allows us to find small proportion differences (between 0.04 and 0.08 approx., depending on the exact proportions) with 85% power and alpha = 0.05, two-tailed. Similarly, trustees (“player B”) were randomly assigned to the time pressure (*n* = 199) or time delay condition (*n* = 202).

The Trust Game was implemented as in Study 2, except for the trustor’s conditions which were conducted using a within-subjects design in Study 3. The players were matched ex-post and trustors did not know which time condition the randomly matched trustee was assigned to. Trustors were informed ex-ante that one half of the trustees (randomly selected) will have to respond under each time condition. To avoid deception, we asked trustors to make one decision for each time condition in random order. The decisions of five (randomly selected) trustees were used to calculate payments for unmatched trustors.

The time conditions were as in Studies 1 and 2. Trustees had to choose between options Y and X either before or after a 10-s timer elapsed, leading to the time pressure and time delay conditions, respectively. Before reaching the decision screen, the trustees did not know the payoffs associated to options Y and X, so that they could not think of their decision in advance. In contrast to the trustors, the trustees did not learn about the existence of another time condition before making their decision. Average (± SD) response time among trustees was 6.42 s (± 4.94) in the time pressure condition and 12.30 s (± 17.78) in the time delay condition. Non-compliance with the time constraint assigned, as in Study 2, implies that the interaction would not take place and both players would earn $0.40 (as if the trustor chose not to trust). This was known in advance by the participants. Like in Study 2 we observed high non-compliance, with 11% and 41% (30% if the “send” click is considered) of the trustees failing to comply in the time pressure and time delay condition, respectively.

After making their decisions, all participants were asked to guess the percentage of players A choosing option L for each time condition (at this time, the trustee learned about the existence of another time condition) and the percentage of player B choosing option X for each time condition, both in random order. Thus, they had to make four guesses. As in Study 2, guesses were implemented in 5% increments from 0 to 100% and participants received $0.10 for each correct guess (i.e., the actual percentage belonged to the interval “guess ± 5%”). Participants were correct only 16% of the time and the earnings were $0.062 (SD 0.078) out of a maximum of $0.40. Finally, the participants had to complete the same post-experimental questionnaire as in Study 2, with identical tasks and protocols, to obtain our battery of control variables. Full instructions can be found in Appendix A3.

### Results study 3: trustors

**Figure 5:**
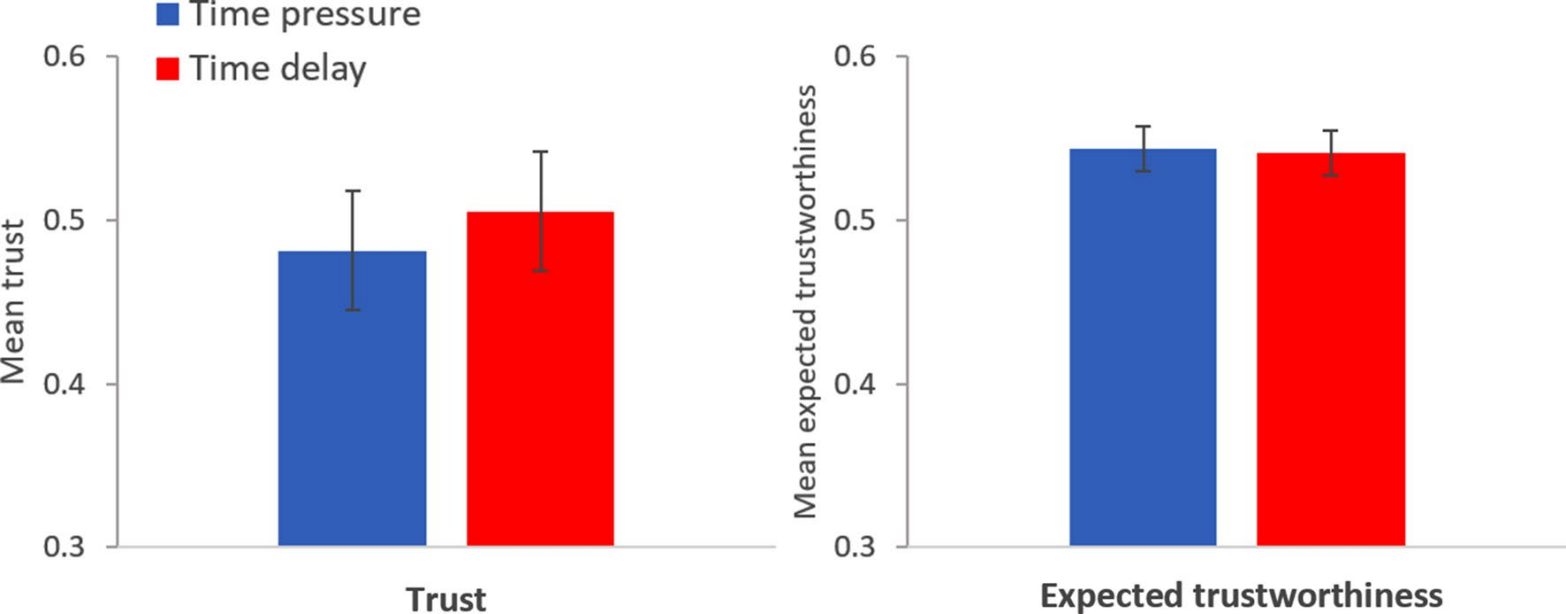
Left panel: Proportion of trustors choosing to trust in the time pressure (blue bar) and time delay condition (red bar). Right panel: Mean trustworthiness expected by trustors in the time pressure (blue bar) and time delay condition (red bar). Error bars represent SE of the difference (within-subjects). Study 3.

Left panel in Fig. 5 displays the proportion (± SE of the difference) of trust choices in each condition. There are no significant differences in trust between the two conditions (one-sample proportion test: proportion difference = 0.024, *p* = 0.51, *n* = 376). Observed proportions: time pressure = 0.481, time delay = 0.505; SE of the difference = 0.036). Given that this is a within-subjects design, we are only interested in testing whether the difference in proportions is significantly different from zero in aggregate terms; hence adding control variables in a regression analysis is not necessary in this case. Thus, we find no support for H1.

Right panel in Fig. 5 displays the mean (± SE of the difference) trustworthiness expected by trustors in each time condition, expressed as the (expected) proportion of trustworthy trustees. There are no significant differences in expected trustworthiness between the two conditions (one-sample t-test: average difference = 0.002, *p* = 0.86, *n* = 376). Observed averages: time pressure = 0.543, time delay = 0.541 (SE of the difference = 0.134). Again, since this is a within-subjects design, we do not conduct regression analysis.

As can be seen in Table S3, however, expected trustworthiness does not significantly predict trust either in the time pressure (mfx of time pressure’s *expected trustworthiness* = 0.107, *p* = 0.31, model 1a) or in the time delay condition (mfx of time delay’s *expected trustworthiness* = 0.147, *p* = 0.18, model 2a). Although the sign of both relationships is positive, this is an unexpected result. In this case, we can test for control variables in the regressions and, in fact, the relationships are slightly stronger when controls are included, but still non-significant for either the time pressure (mfx of time pressure’s *expected trustworthiness* = 0.149, *p* = 0.17, model 1b) or the time delay condition (mfx of time delay’s *expected trustworthiness* = 0.167, *p* = 0.14, model 2b). To further explore this result, we conduct several checks (available upon request). First, we observe that the expected trustworthiness in one condition predicts trust in the same condition slightly better than in the other condition, but always not significantly so, as mentioned. Second, we combine the trustors’ two decisions and set dummy variables for choosing to trust in both decisions (mean proportion = 0.335) and choosing to trust in none of them (mean proportion = 0.348); we also set a variable for the mean expected trustworthiness combining the two conditions (mean = 0.542). From all the possible relationships, the only one that yields *p* < 0.05 is the (negative) relationship between “trust in none” and mean expected trustworthiness when controls are included (without controls: mfx of mean *expected trustworthiness* = − 0.236, *p* = 0.070; with controls: mfx of mean *expected trustworthiness* = − 0.269, *p* = 0.038). Third, we average the two trust decisions (mean = 0.493) and conduct OLS regressions. The relationships between mean trust and mean expected trustworthiness, with controls are included, is not significant (without controls: coeff of *mean expected trustworthiness* = 0.166, *p* = 0.15; with controls: coeff of *mean expected trustworthiness* = 0.202, *p* = 0.073). Thus, we find no support for H2, and expected trustworthiness does not significantly predict trust in either condition separately.

### Results study 3: trustees

**Figure 6:**
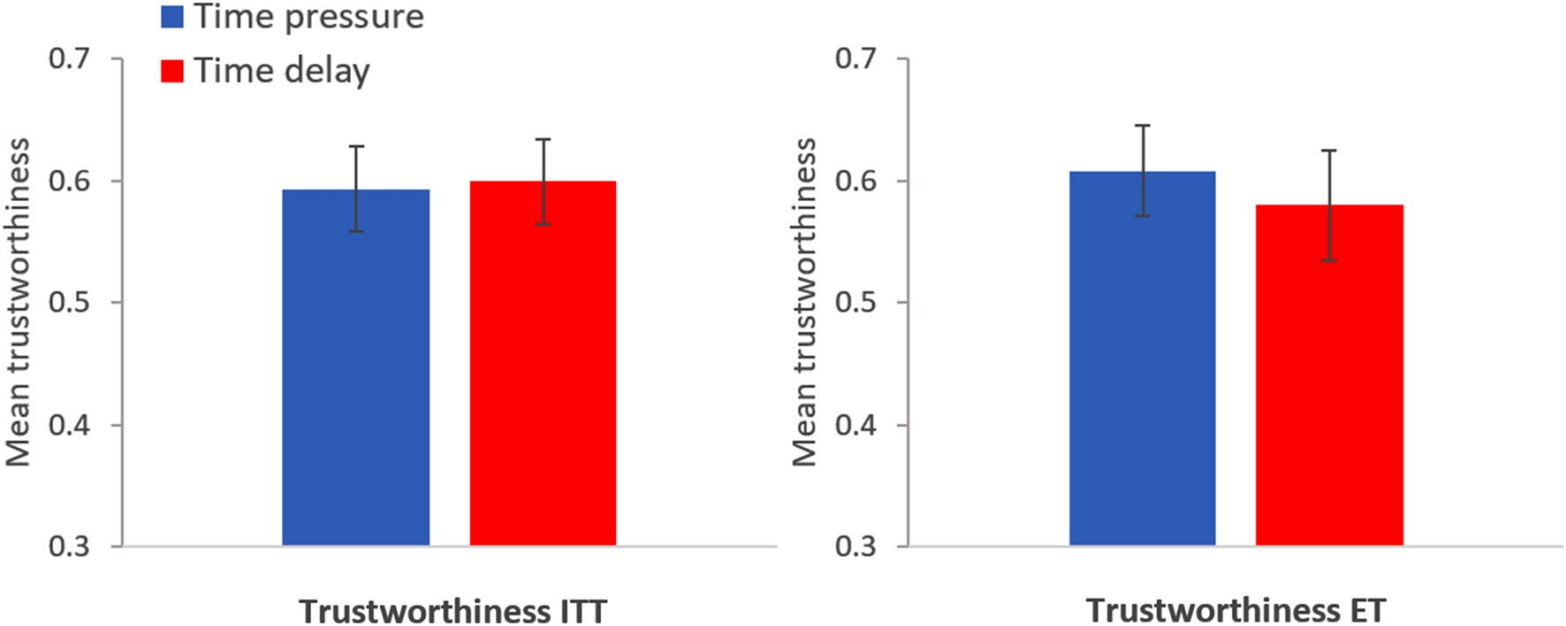
Left panel: Mean proportion of trustworthy trustees in the time pressure (blue bar) and time delay condition (red bar) using an intent-to-treat approach. Right panel: Mean proportion of trustworthy trustees in the time pressure (blue bar) and time delay condition (red bar) using only effectively treated subjects. Error bars represent robust SE. Study 3.

The left panel in Fig. 6 displays the proportion (± SE) of trustworthy trustees in each condition in Study 3 using an intent-to-treat approach (ITT; *n* = 199 for time pressure; *n* = 202 for time delay). There are no significant differences in trustworthiness between the two conditions (mfx of *time delay* = 0.006, *p* = 0.90, *n* = 401). Observed proportions (± SE): time pressure = 0.593 ± 0.035, time delay = 0.599 ± 0.035. The regression analysis can be found in Table S6, model 1a. The right panel in Fig. 6 displays the proportion (± SE) of trustworthy trustees in each condition in Study 3 using only subjects who were effectively treated (ET; *n* = 176 for time pressure; *n* = 119 for time delay). There are no significant differences in trustworthiness between the two conditions (mfx of *time delay* = − 0.028, *p* = 0.63, *n* = 295). Observed proportions (± SE): time pressure = 0.608 ± 0.037, time delay = 0.580 ± 0.045. The regression analysis can be found in Table S6, model 2a. In Table S6, models 1b and 2b, we show that these results are robust to controlling for all the variables considered in this study.

### Discussion of study 3

In Study 3, we again fail to find support for either H1 or H2. In a highly powered within-subjects design, which would presumably be very favorable for any difference to emerge, thus we still find that trustors do not adjust their behavior or beliefs to the trustee’s time conditions. Interestingly, although the expected trustworthiness in one condition predicts trust in the same condition slightly better than in the other condition, this relationship is not significant in either case even when controls are included. To find significant or marginally significant results we need to combine the trust decisions as well as the expected trustworthiness in the two conditions. This suggests that the trustee’s time conditions are nearly irrelevant for the trustors and that observing such conditions can be confusing for them since, even combining both conditions, more expected trustworthiness is only weakly related to trust. In other words, the trustee’s time conditions seem to be more confusing for trustors than meaningful.

## General discussion

Regarding our main hypotheses H1 and H2, according to which trustors should trust more and expect more trustworthiness by trustees who are forced to respond quickly versus slowly, the results are straightforward: across different designs, including both between- and within-subjects analyses as well as both continuous and binary decision spaces, we find that trustors do not adjust their behavior or beliefs (about trustworthiness) to the trustee’s time conditions. Note that some of our analyses were well powered to detect rather small effects and presumably very favorable to the emergence of any existing difference. Thus, H1 and H2 are largely unsupported by the data.

The results regarding trustees’ behavior are more mixed. In the only case in which we find a difference, the data suggests a positive effect of time delay (vs. pressure) on trustworthiness. Note that the finding that forced delay triggers more trustworthiness than time pressure among our trustees in Study 1 might be due to several factors^16,18,29,43,44^, including a lower presence of mistakes^36,46^. In fact, although we allowed subjects to familiarize themselves with the decision slider prior to making their choices and we do not find evidence of greater randomness in trustees’ responses in the time pressure condition (variance-comparison test, f(74, 74) = 1.02, *p* = 0.92), an error-based explanation cannot be completely ruled out. When we set designs that minimize the effect of errors (Study 2 and Study 3), we find no effect at all. Thus, our results provide evidence against the so-called “social heuristics hypothesis” (SHH)^14,15^ and are in line with recent inconclusive or failed replications^31,72^.

However, most previous studies on the SHH do not consider trustworthiness but more “general” cooperation decisions instead. It is surprising that the evidence on the effect of cognitive manipulations (including time constraints) on trustworthiness is so scarce, given that trustworthiness/reciprocity provides for one of the cleanest tests of the SHH^30,39^: it typically pays off in everyday situations but is disadvantageous in one-shot experiments, and it does not depend on beliefs about the other party’s behavior (in contrast to other games such as the prisoners dilemma or public goods game, which have been extensively used in tests of the SHH). The only studies we are aware of that test the effect of cognitive manipulations on trustee’s decisions (using ego depletion^40^, hunger^73^, or time constraints^15^) typically find that intuition promotes trustworthiness, albeit non-significantly in some cases. At the trait level, Corgnet et al.^45^ find that more reflective individuals trust more but they find no relationship between reflectiveness and trustworthiness. Considering the importance of trustworthiness and reciprocity for the SHH, the literature clearly needs to be extended with regards to the effect of cognitive manipulations on trustees’ behavior.

The existence of exogenous time constraints is ubiquitous in many real-life decisions and, therefore, our results have important implications for both the public and the private spheres^22,23^. For example, “cooling-off” periods are often proposed in situations such as negotiations^25,26^, divorce decisions (e.g., in Korea^27^) and consumer purchases^24^ where trust is a key consideration. Furthermore, stock markets across the world have built in circuit breakers in case of unusually large price movements with the aim of downplaying panic selling and other “irrational” patterns (although it remains unclear whether the benefits of such regulatory practices overcome their costs^74–76^). Exploding-offer markets in which there is no time to consider different alternatives are typically inefficient and, thus, the recommendation also tends to be to increase the amount of time to make decisions^21^. Our results suggest that, in contrast to our initial hypothesis, forcing agents to delay their decisions does not reduce others’ trust. This means that cooling-off and similar regulations seem to be safe from potential inefficiencies associated to lack of trust. In sum, our findings indicate that reflective (vs. intuitive) decisions are met with distrust^6,7,12^, if anything, only when the decisions’ character allow to make inferences about the decision maker’s personality, which is not the case when time constraints are externally imposed. That said, our results might not be extended to any real-life situation with time constraints. Our experiments focus on a particularly short time horizon and, even though some of the above real-life examples use similarly short time windows (such as exploding offers in online markets), our findings might not apply to situations with longer time constraints. Analyzing this is left for future studies.

## Supplementary Information

Supplementary Information 1.

Supplementary Information 2.

## Data Availability

All data and code (STATA format) for this study are included in the Supplementary Information files.
