# Supplementary material for: Trustors’ disregard for trustees deciding quickly or slowly in three experiments with time constraints
Source: Sci Rep. 2022 Jul 15;12:12120. doi: 10.1038/s41598-022-15420-2 (PMC9287382; doi:10.1038/s41598-022-15420-2)
Supplement: Supplementary file 1 — Supplementary Information 1. [file 41598_2022_15420_MOESM1_ESM.pdf]

SUPPLEMENTARY MATERIALS

Trustors' disregard for trustees deciding quickly or slowly in three experiments with time constraints

Antonio Cabrales, Antonio M. Espín, Praveen Kujal, & Stephen Rassenti

Supplementary Tables

Table S1. Determinants of trustors' trust (Study 1)

	Model 1a	Model 1b	Model 2a	Model 2b
dep var:	amount trusted		amount trusted 2-5	
tdelay	-0.378	-0.428	0.565**	0.589**
	(0.472)	(0.453)	(0.217)	(0.223)
			0.200	0.206
male		0.185		0.274
		(0.494)		(0.235)
				0.099
CRT score		0.301*		-0.079
		(0.119)		(0.056)
				-0.028
envy		-0.390		0.177
		(0.225)		(0.120)
				0.063
compassion		0.271		0.025
		(0.225)		(0.111)
				0.009
impatience		0.017		-0.017
		(0.045)		(0.023)
				-0.006
risk aversion		-0.080		0.027
		(0.092)		(0.057)
				0.010
Constant	4.919***	4.813***	-0.750***	-1.063
	(0.356)	(1.074)	(0.161)	(0.562)
F/Chi^2	0.641	3.070**	6.751**	13.962
ll	-371.122	-363.372	-91.318	-87.861
R^2/pseudo-R^2	0.004	0.102	0.036	0.073
N	150	150	150	150

Notes: OLS (model 1: amount sent) and probit (model 2: amount sent between 2 and 5) estimates. Robust standard errors in parentheses. Marginal effects are reported below standard errors for probit models. Main explanatory variable: *tdelay* takes the value of 1 for the time delay condition, 0 for the time pressure condition. Controls: *male* takes the value of 1 if male, 0 otherwise; *CRT score* refers to the number of correct answers in the CRT (from 0 to 7); *envy* refers to the number of envious choices in the distributional preferences task (from 0 to 3); *compassion* refers to the number of compassionate choices in the distributional preferences task (from 0 to 3); *impatience* refers to the number of impatient choices in the time preferences task (from 0 to 20); *risk aversion* refers to the number of risk averse choices in the risk preferences task (from 0 to 10). * p<0.05, ** p<0.01, *** p<0.001.

Table S2. Determinants of trustors' trust and beliefs (Study 2)

	Model 1a	Model 1b	Model 2a	Model 2b	Model 3a	Model 3b	Model 4a	Model 4b
dep var:	trust (both conditions)		trust (time pressure)		trust (time delay)		exp trustworthiness	
tdelay	0.031	0.056					-0.020	-0.029
	(0.126)	(0.129)					(0.025)	(0.024)
	0.012	0.022						
exp trustworthiness			1.187**	1.451***	1.128**	1.176**		
			(0.364)	(0.388)	(0.377)	(0.414)		
			0.474	0.579	0.450	0.469		
male		-0.144		-0.378		0.104		0.021
		(0.135)		(0.195)		(0.205)		(0.026)
		-0.057		-0.150		0.041		
age		0.000		-0.006		0.009		-0.000
		(0.006)		(0.008)		(0.009)		(0.001)
		0.000		-0.002		0.003		
CRT score		0.041		0.089		0.044		-0.014*
		(0.032)		(0.048)		(0.046)		(0.006)
		0.016		0.035		0.018		
envy		-0.121		-0.173		-0.059		0.004
		(0.072)		(0.108)		(0.108)		(0.013)
		-0.048		-0.069		-0.023		
compassion		0.179*		0.101		0.172		0.044***
		(0.072)		(0.101)		(0.110)		(0.013)
		0.071		0.040		0.069		
impatience		-0.006		0.019		-0.031*		-0.001
		(0.011)		(0.016)		(0.016)		(0.002)
		-0.002		0.007		-0.013		
risk aversion		-0.087**		-0.067		-0.141**		0.004
		(0.028)		(0.042)		(0.043)		(0.005)
		-0.035		-0.027		-0.056		
loss aversion		-0.003		0.020		0.011		0.002
		(0.043)		(0.064)		(0.064)		(0.008)
		-0.001		0.008		0.004		
Constant	0.006	0.403	-0.613**	-0.424	-0.526*	-0.071	0.520***	0.431***
	(0.089)	(0.393)	(0.207)	(0.582)	(0.206)	(0.603)	(0.018)	(0.076)
F/Chi^2	0.062	22.229**	10.614**	22.177**	8.940**	29.413***	0.650	1.979*
ll	-275.087	-263.040	-131.394	-123.954	-134.070	-122.520	-3.181	5.788
R^2/pseudo-R^2	0.000	0.044	0.038	0.092	0.032	0.116	0.002	0.046
N	397	397	197	197	200	200	397	397

Notes: Probit (models 1-3: trust) and OLS (model 4: beliefs, expected trustworthiness) estimates. Robust standard errors in parentheses. Marginal effects are reported below standard errors for probit regressions. Main explanatory variables: *tdelay* (see notes on Table S1); *exp trustworthiness* refers to the expected proportion of trustworthy trustees in the corresponding condition. Controls: *loss aversion* refers to the number of loss averse choices in the loss aversion task (from 0 to 6). See notes on Table S1 for the remaining controls. * p<0.05, ** p<0.01, *** p<0.001.

Table S3. Determinants of trustors' trust (Study 3)

	Model 1a	Model 1b	Model 2a	Model 2b
dep var:	trust (time pressure)		trust (time delay)	
exp trustworthiness	0.268	0.373	0.369	0.418
	(0.265)	(0.270)	(0.277)	(0.281)
	0.107	0.149	0.147	0.167
male		-0.049		-0.270*
		(0.136)		(0.136)
		-0.019		-0.107
age		0.010		-0.002
		(0.006)		(0.005)
		0.004		-0.001
CRT score		0.037		0.022
		(0.033)		(0.032)
		0.015		0.009
envy		-0.074		-0.012
		(0.070)		(0.070)
		-0.030		-0.005
compassion		0.113		0.126
		(0.071)		(0.072)
		0.045		0.050
impatience		0.016		-0.007
		(0.012)		(0.012)
		0.006		-0.003
risk aversion		-0.071*		-0.056
		(0.033)		(0.033)
		-0.028		-0.022
loss aversion		0.038		0.046
		(0.048)		(0.049)
		0.015		0.018
Constant	-0.193	-0.726	-0.186	-0.069
	(0.158)	(0.424)	(0.163)	(0.406)
Chi^2	1.026	14.393	1.767	13.086
ll	-259.853	-252.720	-259.723	-253.672
pseudo-R^2	0.002	0.029	0.003	0.027
N	376	376	376	376

Notes: Probit estimates (trust). Robust standard errors in parentheses. Marginal effects are reported below standard errors. Main explanatory variable: *exp trustworthiness* refers to the expected proportion of trustworthy trustees in the corresponding condition. Controls: see notes on Tables S1 and S2. * p<0.05, ** p<0.01, *** p<0.001.

Table S4. Determinants of trustees' trustworthiness (Study 1)

	Model 1a	Model 1b	Model 2a	Model 2b
dep var:		amount returned		
tdelay	1.081*	0.961*	-1.029	-0.785
	(0.485)	(0.476)	(0.630)	(0.647)
amount received	0.309***	0.309***	0.245***	0.255***
	(0.027)	(0.028)	(0.038)	(0.040)
tdelay X a received			0.149**	0.126*
			(0.051)	(0.055)
male		-0.206		-0.129
		(0.543)		(0.542)
envy		-0.051		-0.084
		(0.270)		(0.266)
compassion		0.784**		0.692**
		(0.249)		(0.248)
risk aversion		-0.172		-0.170
		(0.118)		(0.118)
impatience		-0.013		-0.028
		(0.056)		(0.056)
Constant	0.251	0.410	1.205*	1.457
	(0.412)	(1.024)	(0.484)	(1.167)
F	73.924***	21.106***	59.134***	21.050***
ll	-376.286	-370.676	-372.747	-368.123
R^2	0.450	0.490	0.476	0.507
N	150	150	150	150
Wald Tests				
a received + tdelay X a received			0.394***	0.381***
			(0.034)	(0.036)
*tdelay + tdelay X a received*3*			-0.580	-0.406
			(0.524)	(0.527)
*tdelay + tdelay X a received*6*			-0.132	-0.027
			(0.446)	(0.438)
*tdelay + tdelay X a received*9*			0.317	0.352
			(0.411)	(0.401)
*tdelay + tdelay X a received*12*			0.765	0.731
			(0.432)	(0.429)
*tdelay + tdelay X a received*15*			1.214*	1.110*
			(0.500)	(0.512)
*tdelay + tdelay X a received*18*			1.662**	1.489*
			(0.601)	(0.629)
*tdelay + tdelay X a received*21*			2.111**	1.867*
			(0.719)	(0.764)
*tdelay + tdelay X a received*24*			2.559**	2.246*
			(0.849)	(0.909)
*tdelay + tdelay X a received*27*			3.008**	2.625*
			(0.986)	(1.060)
*tdelay + tdelay X a received*30*			3.456**	3.004*
			(1.126)	(1.215)

Notes: OLS estimates (amount returned). Robust standard errors in parentheses. Wald Tests on the interaction coefficients: a received + tdelay X a received refers to the effect of amount received in the time delay condition (for the time pressure condition, the effect is given by the coefficient of

amount received); tdelay + tdelay X a received*Z refers to the effect of time delay for an amount received of $Z. Controls: see notes on Table S1. * p<0.05, ** p<0.01, *** p<0.001.

Table S5. Determinants of trustees' trustworthiness (Study 2)

	Model 1a	Model 1b	Model 2a	Model 2b
dep var:	trustworthiness (ITT)		trustworthiness (ET)	
tdelay	0.054	-0.120	0.128	-0.013
	(0.127)	(0.145)	(0.149)	(0.168)
	0.021	-0.046	0.049	-0.005
male		-0.032		0.047
		(0.148)		(0.169)
		-0.013		0.018
age		0.002		0.001
		(0.007)		(0.007)
		0.001		0.000
CRT score		0.030		0.008
		(0.035)		(0.039)
		0.011		0.003
envy		0.088		0.123
		(0.078)		(0.089)
		0.034		0.046
compassion		0.765***		0.783***
		(0.075)		(0.086)
		0.295		0.296
impatience		0.002		-0.010
		(0.012)		(0.013)
		0.001		-0.004
risk aversion		0.045		0.032
		(0.034)		(0.039)
		0.017		0.012
loss aversion		-0.046		-0.040
		(0.049)		(0.055)
		-0.018		-0.015
Constant	0.189*	-1.750***	0.221*	-1.574**
	(0.089)	(0.439)	(0.092)	(0.504)
Chi^2	0.180	113.861***	0.743	90.284***
ll	-269.945	-203.544	-207.422	-156.128
pseudo-R^2	0.000	0.246	0.002	0.249
N	398	398	310	310

Notes: Probit estimates (trustworthiness). Robust standard errors in parentheses. Marginal effects are reported below standard errors. See notes on Tables S1 and S2. * p<0.05, ** p<0.01, *** p<0.001.

Table S6. Determinants of trustees' trustworthiness (Study 3)

	Model 1a	Model 1b	Model 2a	Model 2b
dep var:	trustworthiness (ITT)		trustworthiness (ET)	
tdelay	0.016	0.068	-0.073	-0.037
	(0.127)	(0.139)	(0.150)	(0.168)
	0.006	0.026	-0.028	-0.014
male		-0.181		-0.158
		(0.141)		(0.167)
		-0.069		-0.060
age		0.009		0.010
		(0.007)		(0.008)
		0.003		0.004
CRT score		0.027		0.013
		(0.034)		(0.040)
		0.010		0.005
envy		0.163*		0.256**
		(0.076)		(0.091)
		0.062		0.098
compassion		0.639***		0.705***
		(0.077)		(0.092)
		0.244		0.268
impatience		-0.017		-0.014
		(0.012)		(0.014)
		-0.007		-0.005
risk aversion		0.060		0.044
		(0.032)		(0.041)
		0.023		0.017
loss aversion		-0.018		-0.009
		(0.048)		(0.056)
		-0.007		-0.003
Constant	0.235**	-1.787***	0.274**	-2.064***
	(0.090)	(0.401)	(0.096)	(0.488)
Chi2	0.015	98.169***	0.232	78.776***
ll	-270.506	-214.636	-198.820	-152.171
pseudo-R2	0.000	0.207	0.001	0.235
N	401	401	295	295

Notes: Probit estimates (trustworthiness). Robust standard errors in parentheses. Marginal effects are reported below standard errors. See notes on Tables S1 and S2. * p<0.05, ** p<0.01, *** p<0.001.

Appendix A1. Instructions for Study 1

[screenshots explanations and treatment manipulations in brackets]

INDIVIDUAL A

Screen 1

Welcome

You have been selected at random as an individual A and will be paired with an individual B (also selected at random).

You (individual A) have received a $10 endowment which will be used for decision making in the experiment. Individual B has received a $10 endowment as well. Individual B will keep their initial endowment regardless of the decision either you or they make.

You (individual A) will make your decision first.

Individual B will make their decision after you.

Please familiarize yourself with the slider bar by clicking in the area that says "Click Here". It will be used in the decision making stage.

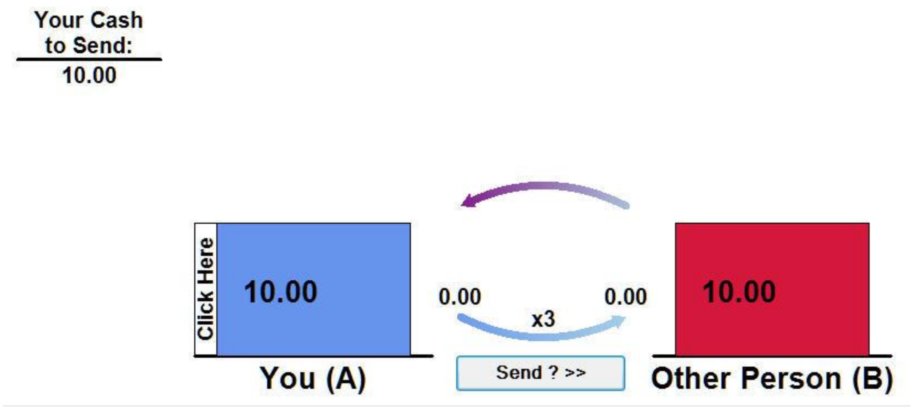

[After clicking on "Click Here". Example of practice decision]

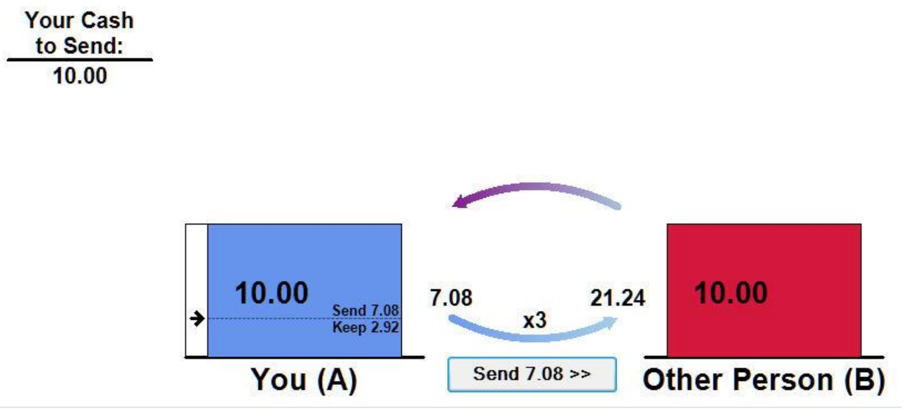

In the experiment today you will interact with the other individual only once. You will not know the identity of the other individual. Similarly, the other individual will not know any details about you. Please do not talk to anyone during the experiment.

Screen 2

The Decision Task

You can transfer any proportion (between $0-$10) of your endowment to individual B.

Individual B will receive 3 times the amount that you transfer (1 becomes 3, 2 becomes 6, and so on.)

Individual B has to decide what proportion of the amount received to return back to you.

Your Profit =

Endowment - (Amount you sent to individual B) + (Amount individual B returns to you)

You will be paid in cash at the end of the experiment.

Individual B can only make their decision **after [before]** 10 seconds has elapsed. That is, after being informed of the amount received, they can only make their final decision **after [before]** 10 seconds has elapsed.

If you have any questions, please raise your hand and a monitor will come by to answer them. If you are finished with the instructions, please click the Start button. The instructions will remain

on your screen until everyone has clicked the Start button. We need *everyone* to click the Start button before we can begin.

[After clicking on “Start”. Example of actual decision]

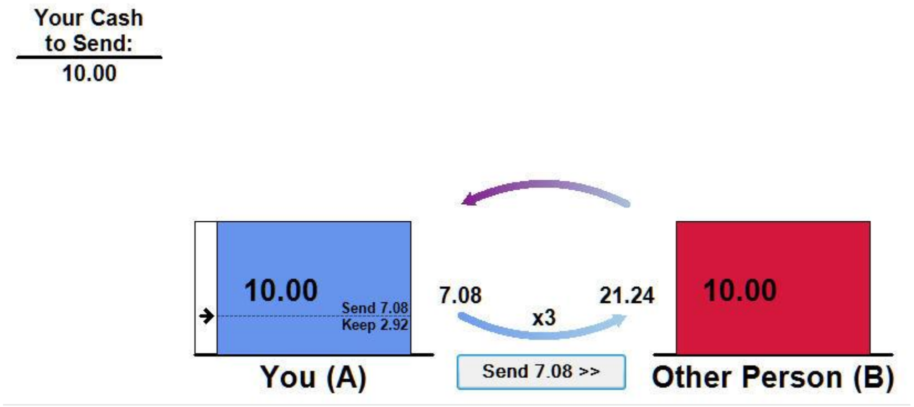

INDIVIDUAL B

Screen 1

Welcome

You have been selected at random as an individual B and will be paired with an individual A (also selected at random).

Individual A has received a $10 endowment which will be used for decision making in the experiment. You (individual B) have received a $10 endowment as well. You will keep your initial endowment regardless of the decision either you or individual A makes.

Individual A will make their decision first.

You (individual B) will make your decision after individual A.

Please familiarize yourself with the slider bar by clicking in the area that says "Click Here". It will be used in the decision making stage.

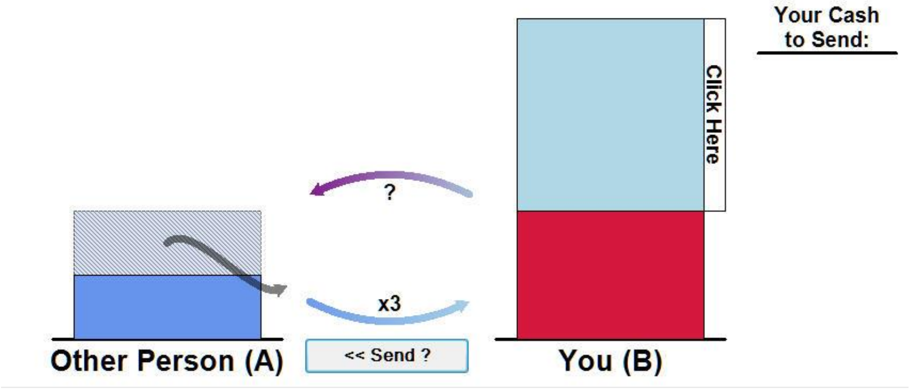

[After clicking on “Click Here”. Example of practice decision]

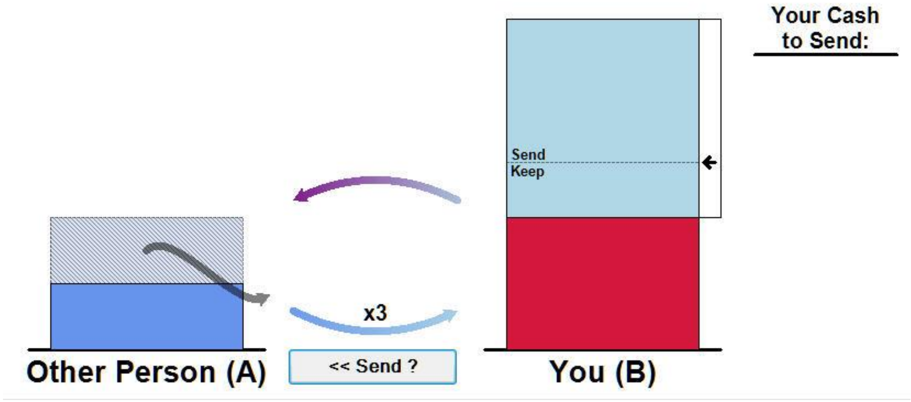

In the experiment today you will interact with the other individual only once. You will not know the identity of the other individual. Similarly, the other individual will not know any details about you. Please do not talk to anyone during the experiment.

Screen 2

The Decision Task

You (individual B) will receive a certain amount of money. This amount is 3 times the amount that individual A sent you.

Individual A can send you any proportion of their $10 endowment and knows that the amount sent is multiplied by 3.

You have to decide how much (between $0 and "the amount received") of this multiplied amount to return to individual A. You can only make your decision **after [before]** a 10 second timer has elapsed.

Your Profit =

Endowment + (Multiplied amount individual A sent to you) - (Amount you returned to individual A).

You will be paid in cash at the end of the experiment.

Please make your decision **after [before]** the 10 second timer has finished.

If you have any questions, please raise your hand and a monitor will come by to answer them. If you are finished with the instructions, please click the Start button. The instructions will remain on your screen until everyone has clicked the Start button. We need *everyone* to click the Start button before we can begin.

[After clicking on "Start". Example of actual decision]

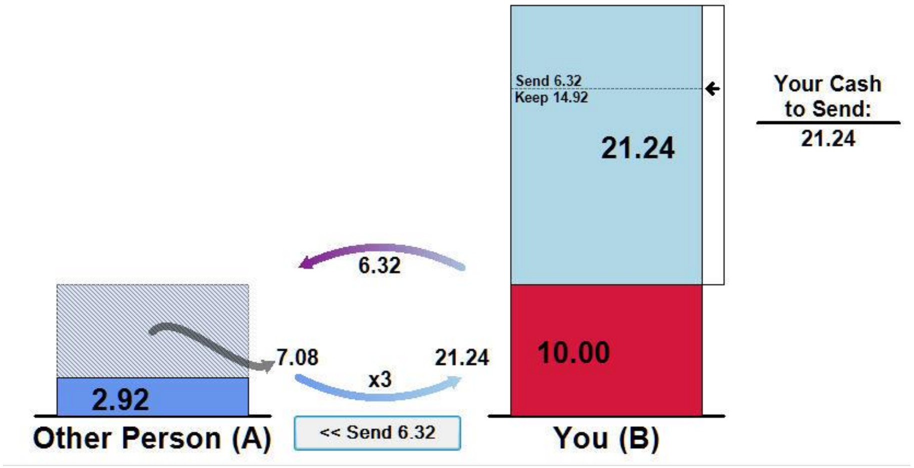

Questionnaire screenshots (identical for all participants)

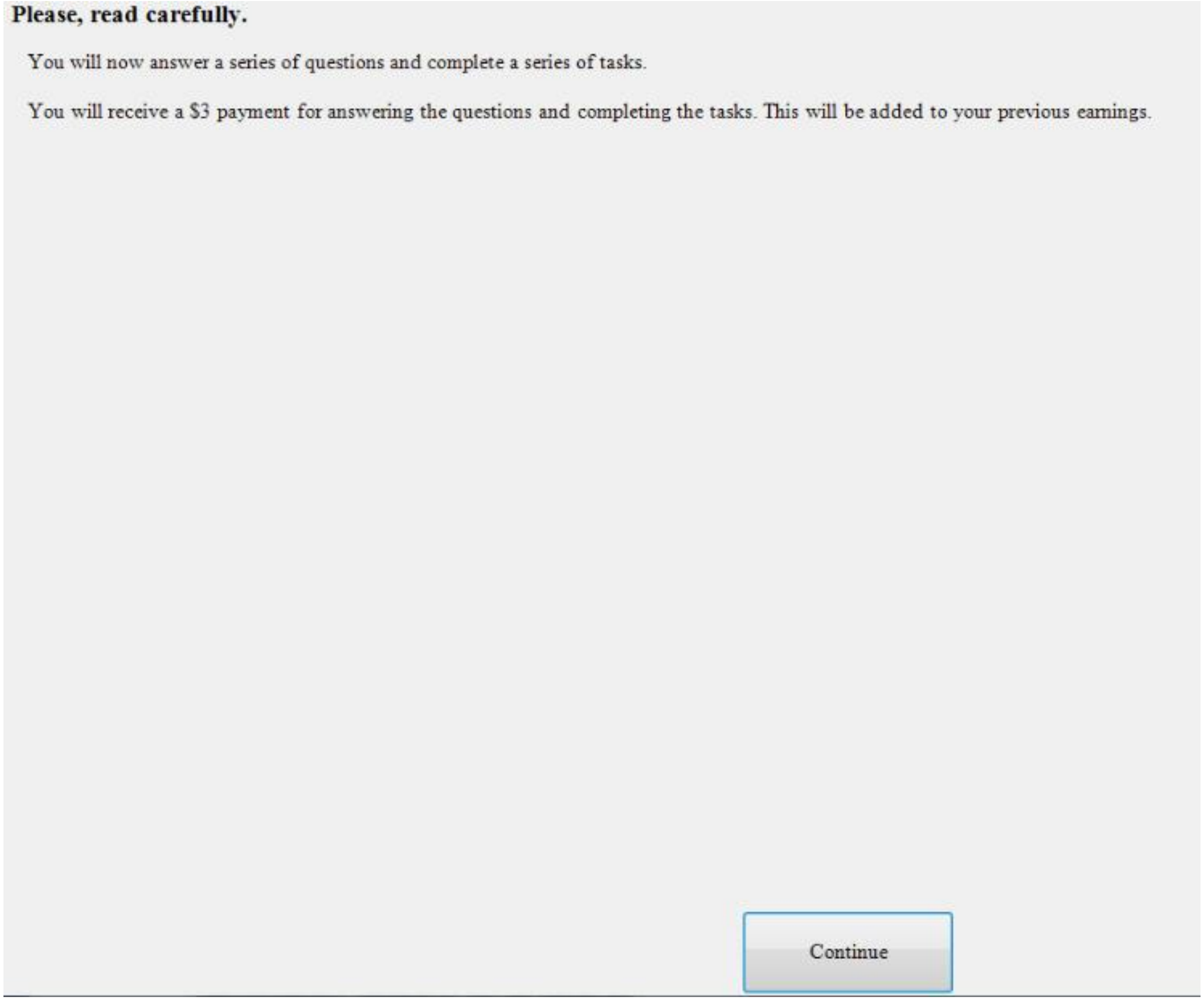

Cognitive Reflection Test

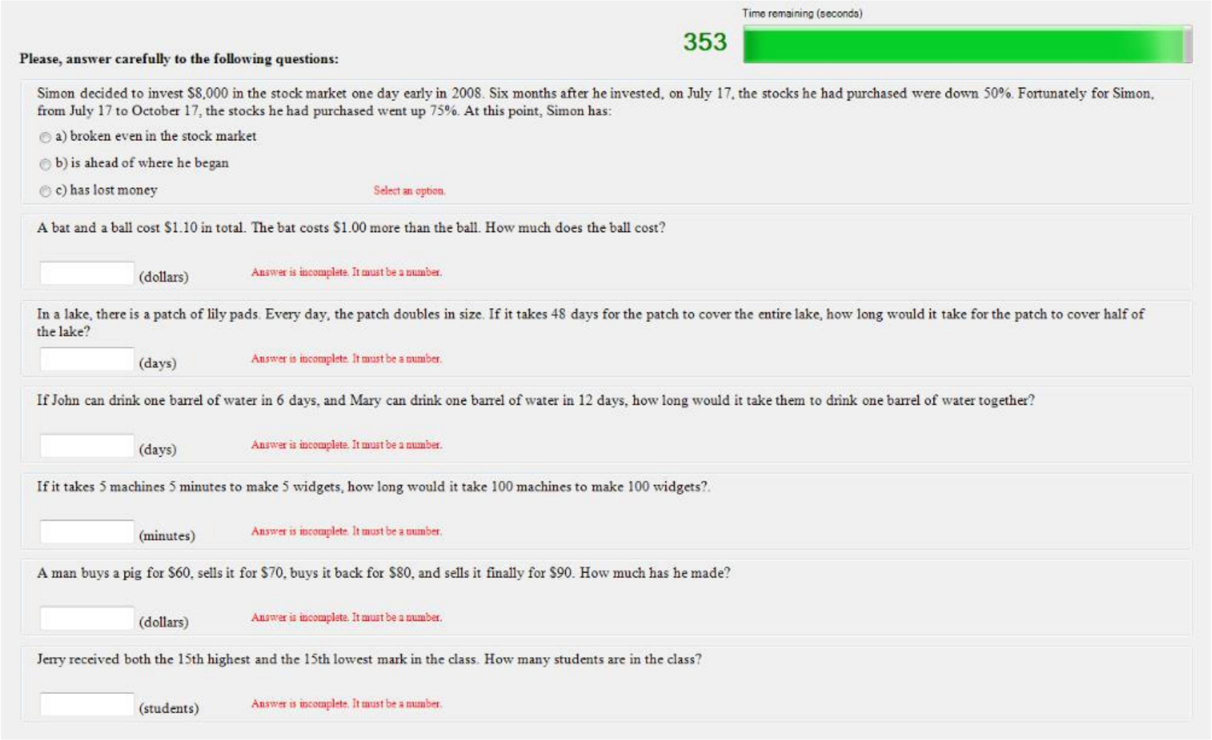

Time remaining (seconds)

353

Please, answer carefully to the following questions:

Simon decided to invest $8,000 in the stock market one day early in 2008. Six months after he invested, on July 17, the stocks he had purchased were down 50%. Fortunately for Simon, from July 17 to October 17, the stocks he had purchased went up 75%. At this point, Simon has:

- a) broken even in the stock market
- b) is ahead of where he began
- c) has lost money

Select an option.

A bat and a ball cost $1.10 in total. The bat costs $1.00 more than the ball. How much does the ball cost?

(dollars) Answer is incomplete. It must be a number.

In a lake, there is a patch of lily pads. Every day, the patch doubles in size. If it takes 48 days for the patch to cover the entire lake, how long would it take for the patch to cover half of the lake?

(days) Answer is incomplete. It must be a number.

If John can drink one barrel of water in 6 days, and Mary can drink one barrel of water in 12 days, how long would it take them to drink one barrel of water together?

(days) Answer is incomplete. It must be a number.

If it takes 5 machines 5 minutes to make 5 widgets, how long would it take 100 machines to make 100 widgets?.

(minutes) Answer is incomplete. It must be a number.

A man buys a pig for $60, sells it for $70, buys it back for $80, and sells it finally for $90. How much has he made?

(dollars) Answer is incomplete. It must be a number.

Jerry received both the 15th highest and the 15th lowest mark in the class. How many students are in the class?

(students) Answer is incomplete. It must be a number.

Risk preferences task

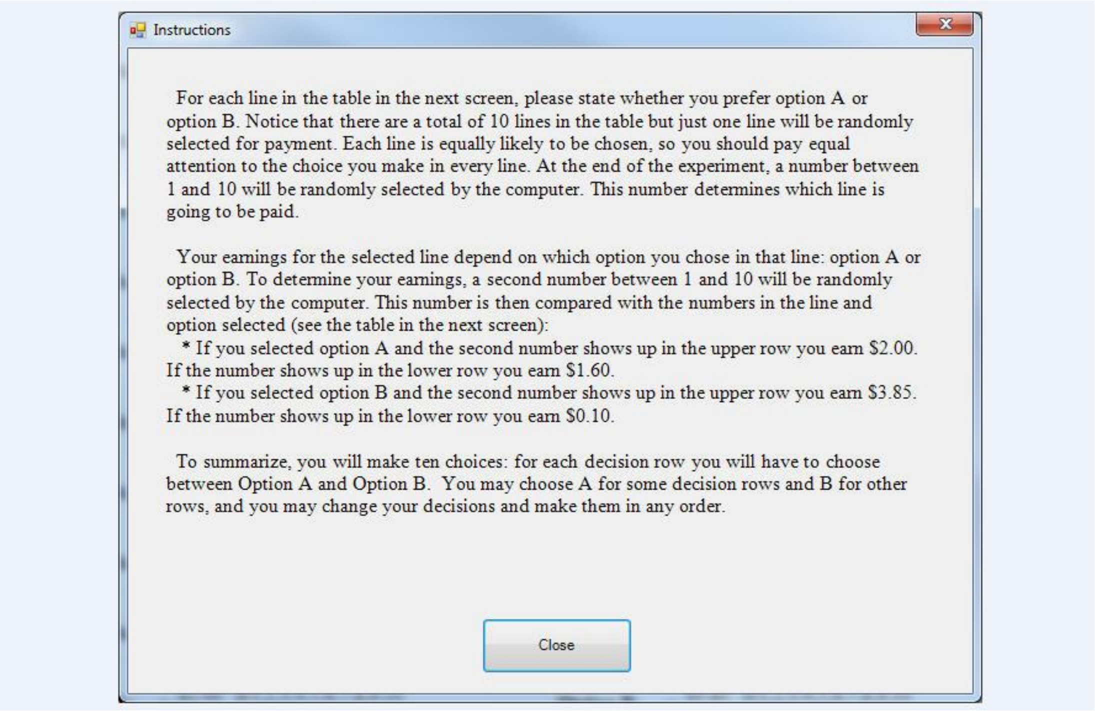
Instructions

For each line in the table in the next screen, please state whether you prefer option A or option B. Notice that there are a total of 10 lines in the table but just one line will be randomly selected for payment. Each line is equally likely to be chosen, so you should pay equal attention to the choice you make in every line. At the end of the experiment, a number between 1 and 10 will be randomly selected by the computer. This number determines which line is going to be paid.

Your earnings for the selected line depend on which option you chose in that line: option A or option B. To determine your earnings, a second number between 1 and 10 will be randomly selected by the computer. This number is then compared with the numbers in the line and option selected (see the table in the next screen):

* If you selected option A and the second number shows up in the upper row you earn $2.00. If the number shows up in the lower row you earn $1.60.
* If you selected option B and the second number shows up in the upper row you earn $3.85. If the number shows up in the lower row you earn $0.10.

To summarize, you will make ten choices: for each decision row you will have to choose between Option A and Option B. You may choose A for some decision rows and B for other rows, and you may change your decisions and make them in any order.

Close

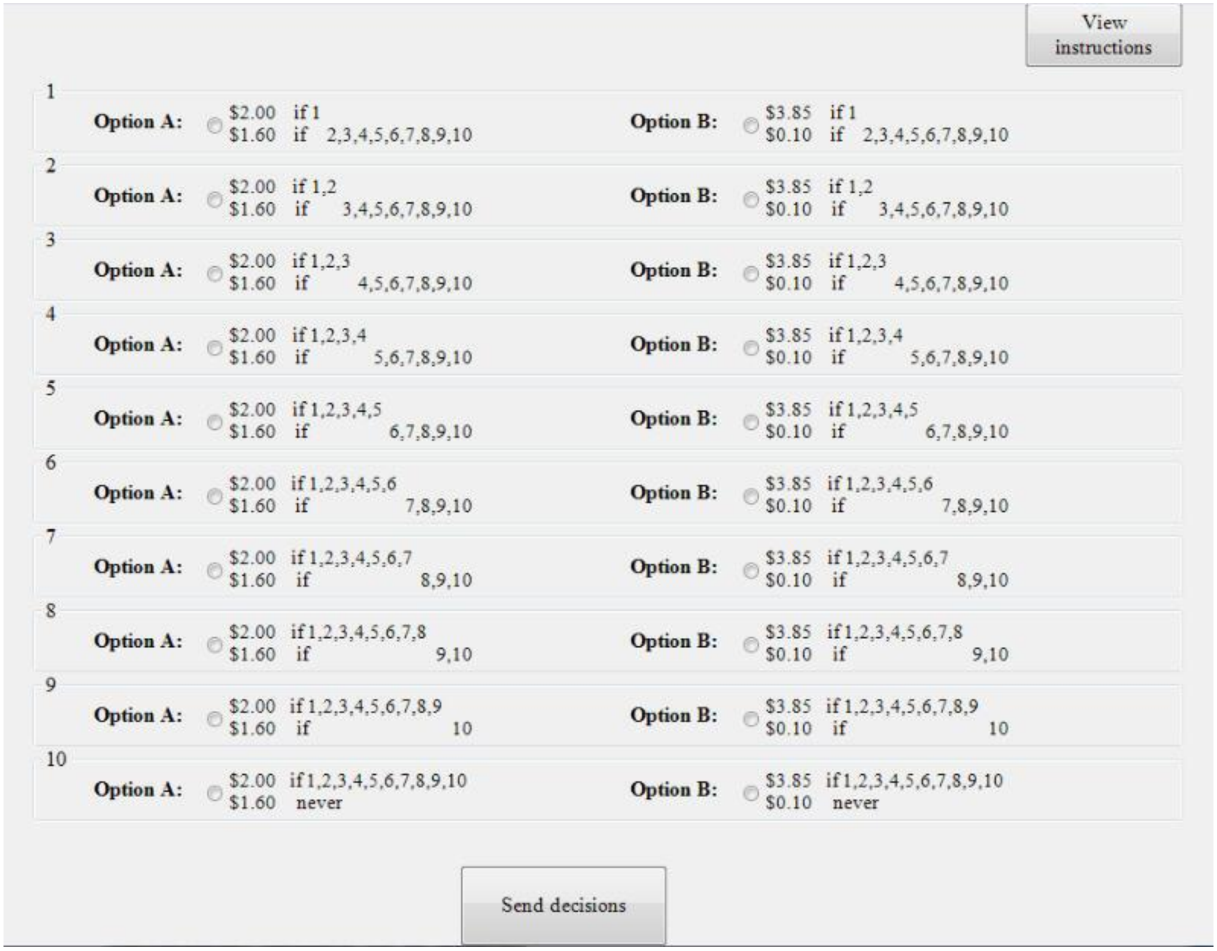
View instructions

1	**Option A:**	$2.00 if 1<br>$1.60 if 2,3,4,5,6,7,8,9,10	**Option B:**	$3.85 if 1<br>$0.10 if 2,3,4,5,6,7,8,9,10
2	**Option A:**	$2.00 if 1,2<br>$1.60 if 3,4,5,6,7,8,9,10	**Option B:**	$3.85 if 1,2<br>$0.10 if 3,4,5,6,7,8,9,10
3	**Option A:**	$2.00 if 1,2,3<br>$1.60 if 4,5,6,7,8,9,10	**Option B:**	$3.85 if 1,2,3<br>$0.10 if 4,5,6,7,8,9,10
4	**Option A:**	$2.00 if 1,2,3,4<br>$1.60 if 5,6,7,8,9,10	**Option B:**	$3.85 if 1,2,3,4<br>$0.10 if 5,6,7,8,9,10
5	**Option A:**	$2.00 if 1,2,3,4,5<br>$1.60 if 6,7,8,9,10	**Option B:**	$3.85 if 1,2,3,4,5<br>$0.10 if 6,7,8,9,10
6	**Option A:**	$2.00 if 1,2,3,4,5,6<br>$1.60 if 7,8,9,10	**Option B:**	$3.85 if 1,2,3,4,5,6<br>$0.10 if 7,8,9,10
7	**Option A:**	$2.00 if 1,2,3,4,5,6,7<br>$1.60 if 8,9,10	**Option B:**	$3.85 if 1,2,3,4,5,6,7<br>$0.10 if 8,9,10
8	**Option A:**	$2.00 if 1,2,3,4,5,6,7,8<br>$1.60 if 9,10	**Option B:**	$3.85 if 1,2,3,4,5,6,7,8<br>$0.10 if 9,10
9	**Option A:**	$2.00 if 1,2,3,4,5,6,7,8,9<br>$1.60 if 10	**Option B:**	$3.85 if 1,2,3,4,5,6,7,8,9<br>$0.10 if 10
10	**Option A:**	$2.00 if 1,2,3,4,5,6,7,8,9,10<br>$1.60 never	**Option B:**	$3.85 if 1,2,3,4,5,6,7,8,9,10<br>$0.10 never

Send decisions

Time preferences task (block 1; block 2 is identical but delays are one month vs. three months)

Instructions

In this task, we ask you to think of an hypothetical situation (you will not be paid the corresponding amount) in which you have to choose between payments in different moments of time.

For each of the following pairs, you have to choose between one of two possible options.

◉ Receive $30 today	○ Receive $30 in one month
◉ Receive $30 today	○ Receive $32 in one month
◉ Receive $30 today	○ Receive $34 in one month
◉ Receive $30 today	○ Receive $36 in one month
◉ Receive $30 today	○ Receive $38 in one month
◉ Receive $30 today	○ Receive $40 in one month
◉ Receive $30 today	○ Receive $42 in one month
◉ Receive $30 today	○ Receive $44 in one month
◉ Receive $30 today	○ Receive $46 in one month
◉ Receive $30 today	○ Receive $48 in one month

Continue

Distributional social preferences task

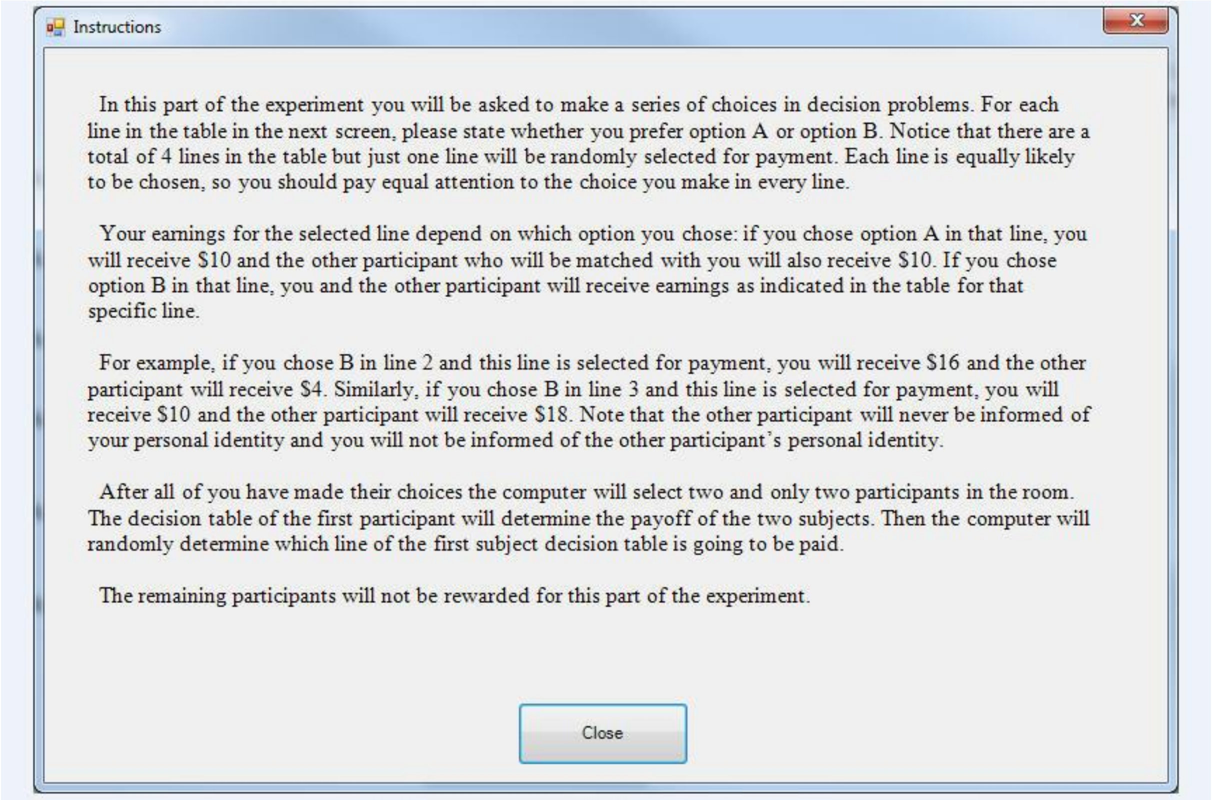

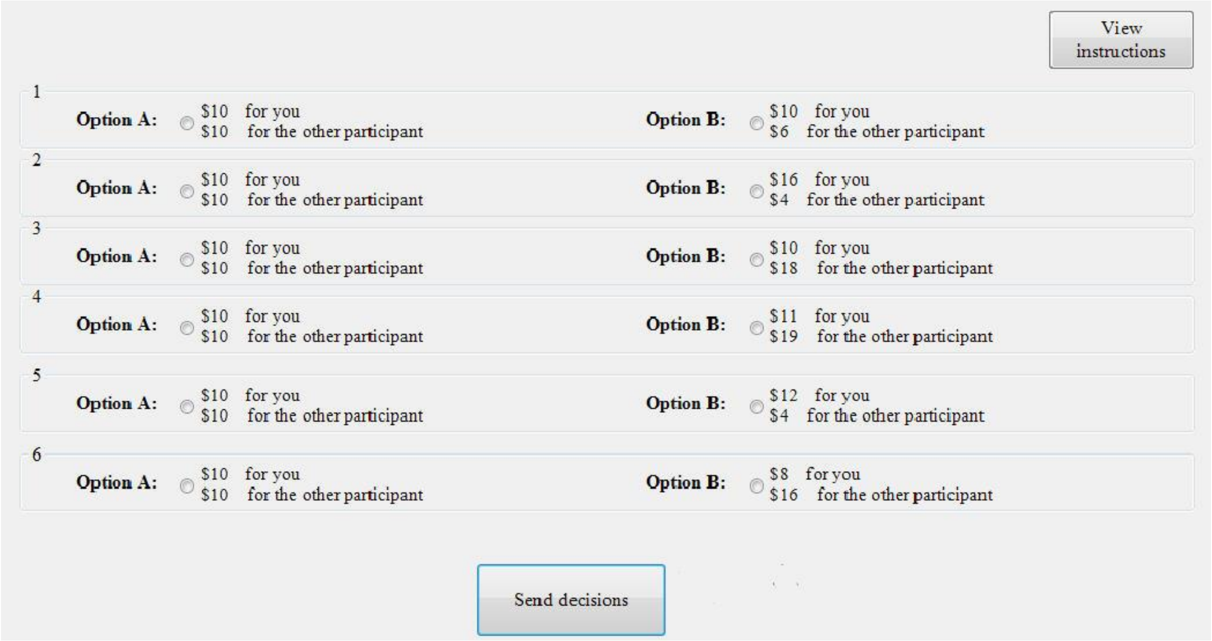

Appendix A2. Instructions for Study 2

PLAYER A

In this task, there are two players, player A and player B. The computer has randomly assigned **you to be player A** and the *other player* you will be matched with will be player B.

You will have to choose between two options:

- If you choose option R then the task ends and you will earn 40¢ and the *other player* will earn 40¢.
- If you choose option L, then the *other player* will have to choose between two options:
    - If the *other player* chooses option Y, then you will earn 0¢ and the *other player* will earn 160¢.
    - If the *other player* chooses option X, then you will earn 80¢ and the *other player* will earn 80¢.

The individual assigned to be **PLAYER B** will have to choose between options Y and X **BEFORE/AFTER** a 10-second timer expires. In case player B does not comply with the time constraint, then both of you will earn 40¢.

In particular, player B will see the following:

{ [instructions for the other person, player B]

In this task, there are two players, player A and player B. The computer has randomly assigned **you to be player B** and the *other player* you will be matched with will be player A.

The other player (A) has to choose between two options:

- If the *other player* chooses option R then the task ends and you will earn 40¢ and the *other player* will earn 40¢.
- If the *other player* chooses option L, then you will have to choose between two options.

For the case that the player A who is matched with you chooses option L, in the next screen, please choose between option Y and option X **BEFORE/AFTER** the 10-second timer in your screen expires (the time that counts is when you click on the option chosen, not on the Continue button).

Note: If you fail to comply with the time constraint, both of you will earn 40¢.

[next screen]

Please choose BEFORE/AFTER the time expires:

- Option Y: you will earn 160¢ and the *other player* will earn 0¢.
- Option X: you will earn 80¢ and the *other player* will earn 80¢.

[10-second TIMER] }

Remember:

- If you choose option R then the task ends and you will earn 40¢ and the *other player* will earn 40¢.
- If you choose option L, then the *other player* will have to choose between two options:
    - If the *other player* chooses option Y, then you will earn 0¢ and the *other player* will earn 160¢.
    - If the *other player* chooses option X, then you will earn 80¢ and the *other player* will earn 80¢.

Now please make your decision:

- You are matched with a player B who has to choose **before/after** the 10-second timer expires:
    - you choose option R
    - you choose option L

<u>BELIEFS (common to both players)</u>

In this task, you will have to guess which percentage of participants choose each option in this experiment. If the difference between your guess and the true percentage is less than 5%, your guess will be considered “correct”. You will be asked for 2 guesses. You will earn 10c extra for each correct guess.

[next screen]

Remember:

Player A has to choose between two options:

- If player A chooses option R then the task ends and player A will earn 40¢ and player B will earn 40¢.
- If player A chooses option L, then player B will have to choose between two options:
    - If player B chooses option Y, then player A will earn 0¢ and player B will earn 160¢.
    - If player B chooses option X, then player A will earn 80¢ and player B will earn 80¢.

Considering that **player B's** have to choose **before/after** the 10-second timer expires

- Among all **PLAYER A's**, you have to guess the percentage of them who chose **OPTION L**.
  Your guess is:
  [slider from 0% to 100% in 5% intervals]
- Among all **PLAYER B's**, you have to guess the percentage of them who chose **OPTION X**.
  Your guess is:
  [slider from 0% to 100% in 5% intervals]

PLAYER B

In this task, there are two players, player A and player B. The computer has randomly assigned **you to be player B** and the *other player* you will be matched with will be player A.

The other player (A) has to choose between two options:

- If the *other player* chooses option R then the task ends and you will earn 40¢ and the *other player* will earn 40¢.
- If the *other player* chooses option L, then you will have to choose between two options.

For the case that the player A who is matched with you chooses option L, in the next screen, please choose between option Y and option X **BEFORE/AFTER** the 10-second timer in your screen expires (the time that counts is when you click on the option chosen, not on the Continue button).

Note: If you fail to comply with the time constraint, both of you will earn 40¢.

[next screen]

Please choose BEFORE/AFTER the time expires:

  - Option Y: you will earn 160¢ and the *other player* will earn 0¢.
  - Option X: you will earn 80¢ and the *other player* will earn 80¢.

[10-second TIMER]

Appendix A3. Instructions for Study 3

PLAYER A

In this task, there are two players, player A and player B. The computer has randomly assigned **you to be player A** and the *other player* you will be matched with will be player B.

You will have to choose between two options:

- If you choose option R then the task ends and you will earn 40¢ and the *other player* will earn 40¢.
- If you choose option L, then the *other player* will have to choose between two options:
    - If the *other player* chooses option Y, then you will earn 0¢ and the *other player* will earn 160¢.
    - If the *other player* chooses option X, then you will earn 80¢ and the *other player* will earn 80¢.

One half of all individuals assigned to be **player B** (selected randomly) will have to choose between options Y and X **before** a 10-second timer expires, while the other half of player Bs will have to choose between Y and X **after** a 10-second timer expires. In case player B does not comply with the time constraint assigned, then both of you will earn 40¢.

In particular, player B will see the following (they will see either "**BEFORE**" or "**AFTER**" below depending on the time constraint they are randomly assigned):

{ [instructions for the other person, player B]

In this task, there are two players, player A and player B. The computer has randomly assigned **you to be player B** and the *other player* you will be matched with will be player A.

The other player (A) has to choose between two options:

- If the *other player* chooses option R then the task ends and you will earn 40¢ and the *other player* will earn 40¢.
- If the *other player* chooses option L, then you will have to choose between two options.

For the case that the player A who is matched with you chooses option L, in the next screen, please choose between option Y and option X **BEFORE/AFTER** the 10-second timer in your screen expires (the time that counts is when you click on the option chosen, not on the Continue button).

Note: If you fail to comply with the time constraint, both of you will earn 40¢.

[next screen]

Please choose BEFORE/AFTER the time expires:

- Option Y: you will earn 160¢ and the *other player* will earn 0¢.
- Option X: you will earn 80¢ and the *other player* will earn 80¢.

[10-second TIMER] }

Since you will be randomly matched with one and only one player B, you will have to choose option R or L for the two possible cases (that is, for the two possible time constraints). Remember:

- If you choose option R then the task ends and you will earn 40¢ and the *other player* will earn 40¢.
- If you choose option L, then the *other player* will have to choose between two options:
  - If the *other player* chooses option Y, then you will earn 0¢ and the *other player* will earn 160¢.
  - If the *other player* chooses option X, then you will earn 80¢ and the *other player* will earn 80¢.

Now please make your decisions:

- If you are matched with a player B who has to choose **before** the 10-second timer expires:
  - you choose option R
  - you choose option L
- If you are matched with a player B who has to choose **after** the 10-second timer expires:
  - you choose option R
  - you choose option L

<u>BELIEFS (common to both players)</u>

In this task, you will have to guess which percentage of participants choose each option in this experiment. If the difference between your guess and the true percentage is less than 5%, your guess will be considered “correct”. You will be asked for 4 guesses. You will earn 10c extra for each correct guess.

[next screen]

Remember:

Player A has to choose between two options:

- If player A chooses option R then the task ends and player A will earn 40¢ and player B will earn 40¢.
- If player A chooses option L, then player B will have to choose between two options:
    - If player B chooses option Y, then player A will earn 0¢ and player B will earn 160¢.
    - If player B chooses option X, then player A will earn 80¢ and player B will earn 80¢.

Among all **player A** individuals, who have to make a choice for two possible time constraints of player B, you have to guess the percentage of them who choose **option L.**

- If player B has to choose **before** the 10-second timer expires, you guess that the percentage of **player A who choose option L** is:
  [slider from 0% to 100% in 5% intervals]
- If player B has to choose **after** the 10-second timer expires, you guess that the percentage of **player A who choose option L** is:
  [slider from 0% to 100% in 5% intervals]

Among all **player B** individuals who choose under each time constraint, you have to guess the percentage of them who choose **option X.**

- If player B have to choose **before** the 10-second timer expires, you guess that the percentage of **player B who choose option X** is:
  [slider from 0% to 100% in 5% intervals]
- If player B have to choose **after** the 10-second timer expires, you guess that the percentage of **player B who choose option X** is:
  [slider from 0% to 100% in 5% intervals]

PLAYER B

In this task, there are two players, player A and player B. The computer has randomly assigned **you to be player B** and the *other player* you will be matched with will be player A.

The other player (A) has to choose between two options:

- If the *other player* chooses option R then the task ends and you will earn 40¢ and the *other player* will earn 40¢.
- If the *other player* chooses option L, then you will have to choose between two options.

For the case that the player A who is matched with you chooses option L, in the next screen, please choose between option Y and option X **BEFORE/AFTER** the 10-second timer in your screen expires (the time that counts is when you click on the option chosen, not on the Continue button).

Note: If you fail to comply with the time constraint, both of you will earn 40¢.

[next screen]

Please choose BEFORE/AFTER the time expires:

  - Option Y: you will earn 160¢ and the *other player* will earn 0¢.
  - Option X: you will earn 80¢ and the *other player* will earn 80¢.

[10-second TIMER]
